# Fitness consequences of polymorphic inversions in the zebra finch genome

**DOI:** 10.1186/s13059-016-1056-3

**Published:** 2016-09-29

**Authors:** Ulrich Knief, Georg Hemmrich-Stanisak, Michael Wittig, Andre Franke, Simon C. Griffith, Bart Kempenaers, Wolfgang Forstmeier

**Affiliations:** 1 Department of Behavioural Ecology and Evolutionary Genetics, Max Planck Institute for Ornithology, 82319 Seewiesen, Germany; 2 Current address: Division of Evolutionary Biology, Faculty of Biology, Ludwig Maximilian University of Munich, 82152 Planegg-Martinsried, Germany; 3 Institute of Clinical Molecular Biology, Christian-Albrechts-University, 24105 Kiel, Germany; 4 Department of Biological Sciences, Macquarie University, Sydney, NSW 2109 Australia; 5 School of Biological, Earth & Environmental Sciences, University of New South Wales, Sydney, NSW 2057 Australia

**Keywords:** Structural variant, Inversion polymorphism, Overdominance, Heterosis, Embryo mortality, Miscarriage

## Abstract

**Background:**

Inversion polymorphisms constitute an evolutionary puzzle: they should increase embryo mortality in heterokaryotypic individuals but still they are widespread in some taxa. Some insect species have evolved mechanisms to reduce the cost of embryo mortality but humans have not. In birds, a detailed analysis is missing although intraspecific inversion polymorphisms are regarded as common. In Australian zebra finches (*Taeniopygia guttata*), two polymorphic inversions are known cytogenetically and we set out to detect these two and potentially additional inversions using genomic tools and study their effects on embryo mortality and other fitness-related and morphological traits.

**Results:**

Using whole-genome SNP data, we screened 948 wild zebra finches for polymorphic inversions and describe four large (12–63 Mb) intraspecific inversion polymorphisms with allele frequencies close to 50 %. Using additional data from 5229 birds and 9764 eggs from wild and three captive zebra finch populations, we show that only the largest inversions increase embryo mortality in heterokaryotypic males, with surprisingly small effect sizes. We test for a heterozygote advantage on other fitness components but find no evidence for heterosis for any of the inversions. Yet, we find strong additive effects on several morphological traits.

**Conclusions:**

The mechanism that has carried the derived inversion haplotypes to such high allele frequencies remains elusive. It appears that selection has effectively minimized the costs associated with inversions in zebra finches. The highly skewed distribution of recombination events towards the chromosome ends in zebra finches and other estrildid species may function to minimize crossovers in the inverted regions.

**Electronic supplementary material:**

The online version of this article (doi:10.1186/s13059-016-1056-3) contains supplementary material, which is available to authorized users.

## Background

Between-individual genetic variation is the substrate for selection. Genetic polymorphisms range in size from single nucleotides (SNPs) to large scale insertions, deletions, or rearrangements that span several millions of base pairs [1, 2]. Among these structural variants, inversions play a prominent role and have long been recognized as drivers of local adaptation and speciation (reviewed in [3]). Inversions are intrachromosomal structural mutations which result in the reversal of gene order (and no change in the genic content of a chromosome) [4].

In heterokaryotypic individuals (those that are heterozygous for an inversion) recombination within the inverted region is largely suppressed, either because homologous pairing is partially inhibited or because crossovers give rise to unbalanced gametes (carrying deletions or duplications) which will lead to the death of the zygote [1]. These two processes are not mutually exclusive and their prevalence depends, amongst others, on the size and location of the inverted region [5–8]. In particular, a distinction between those inversions which cover both chromosome arms and thus include the centromere (pericentric inversions) and those which are restricted to a single chromosome arm (paracentric inversions) has often been made [9]. A single crossover within a pericentric inversion leads to the formation of two chromatids with duplications and deficiencies and two normal chromatids, whereas in paracentric inversions an acentric fragment and a dicentric chromatid along with two normal chromatids are formed [9]. In species with an ordered (linear) tetrad in the female meiosis [e.g., *Drosophila* spp. or maize (*Zea mays*)] paracentric inversions often do not cause reduced fertility in females because the dicentric chromatid is preferentially passed into the second polar body [6, 9]. On the other hand, pericentric inversions often lead to decreased fertility in females [6, 7], which may also explain the preponderance of polymorphic paracentric over pericentric inversions in species like *Drosophila* spp. that lack male recombination [10]. In contrast, humans and maize recombine in the male meiosis and heterokaryotypic males for both pericentric and paracentric inversions may produce a high percentage of unbalanced gametes and hence inviable embryos [5, 11–14], with recombination frequency being highest in the largest inversions (absolute or proportional to the total chromosome size).

Despite their presumed heterozygous fitness costs, which would ultimately lead to the loss of the minor allele from the population, inversion polymorphisms are ubiquitously found within species (reviewed in [3], [15–22]). Inversion polymorphisms are, therefore, somewhat of an evolutionary puzzle. In order to increase in allele frequency, the inverted region should either confer a fitness advantage to the organism or exhibit segregation distortion (drift as the sole force is unlikely but may contribute in small populations [23]). The most prominent feature of an inversion is its ability to suppress recombination within the inverted region. This may preserve linkage disequilibrium between beneficial combinations of alleles, which could lead to the spread of the inversion (with epistatic fitness interactions [24] or without epistasis [25, 26]).

Once a beneficial inversion starts to spread in a population, several mechanisms may prevent it from going to fixation, thereby maintaining the polymorphic state at some equilibrium frequency. The frequencies of most of the known inversion polymorphisms within a species vary latitudinally [18, 27–30], locally [21], or seasonally [31], apparently in response to a changing environment. However, there are also examples of polymorphisms within single populations which could be stabilized via frequency-dependent (disruptive) selection [17, 32–34], antagonistic pleiotropy [35], mate choice [36, 37], recessive deleterious mutations captured by or accumulating on the inverted haplotype (“associative overdominance” [23, 38]), overdominance (i.e., the heterokaryotypic individuals have higher fitness than both homozygotes [38, 39]), or under several scenarios involving segregation distortion [40, 41]. Several of these scenarios will effectively result in overdominance for fitness or in fitness being negatively correlated with the inversion’s frequency; both of which should be possible to measure empirically.

In birds, intraspecific inversion polymorphisms are regarded as common [42, 43], yet it is unknown whether birds have evolved mechanisms to suppress recombination within inversions to reduce the cost of embryo mortality. The two best-studied cases are found in ruffs (*Philomachus pugnax*) and in white-throated sparrows (*Zonotrichia albicollis*). In both species an inversion is linked to distinct plumage and behavioral phenotypes, covering around 100 and 1000 genes, respectively. By (almost) completely suppressing recombination between arrangements these inversions constitute so-called supergenes [19, 20, 22, 44]. In white-throated sparrows, the inversion likely became polymorphic through a past hybridization event [22] and is kept polymorphic by disassortative mating between birds with the two arrangements (e.g., [22, 37, 44]).

The zebra finch (*Taeniopygia guttata*) belongs to the family of grassfinches (Estrildidae), which are rich in polymorphic inversions [45–47]. Two polymorphic pericentric inversions have been described cytogenetically in zebra finches, one on chromosome *Tgu5* (the sixth largest chromosome in the karyotype [45, 48, 49]) and one on the sex chromosome *TguZ* [50]. The inversion polymorphism on chromosome *TguZ* is found in the Australian subspecies (*T. guttata castanotis*) and, with a different allele frequency, in the subspecies from Timor (*T. guttata guttata*) [50].

Here we report on a genome-wide scan for inversion polymorphisms in a wild population of 948 zebra finches from Australia. Due to their nomadic behavior, Australian zebra finches appear to form one very large panmictic population [51, 52], such that the sampled birds are considered representative for the entire Australian subspecies. Using 4553 SNPs, we searched for unusual patterns of long-range linkage disequilibrium and identified four large linkage blocks (two of which are the known inversion polymorphisms on chromosomes *Tgu5* and *TguZ*). We then inferred the inversion genotypes for every individual by principle component analysis, selected unique tagging SNPs, and genotyped an additional set of 5229 birds from four different captive populations of Australian zebra finches. We used these data to study the phenotypic and fitness consequences of the four inversion polymorphisms. Our aim was to address three main issues. (1) Heterokaryotypic individuals should exhibit increased embryo mortality rates if they are unable to completely suppress recombination within the inverted region or to remove the unbalanced meiotic products. We test this prediction by analyzing the occurrence of natural embryonic deaths in 9764 developing eggs. (2) Heterosis and frequency-dependent selection could balance inversion polymorphisms; we test both selective forces by correlating inversion genotypes with several fitness parameters (viability, fecundity, siring success, and number of independent offspring). (3) Effect sizes of associations between inversion genotypes and polygenic traits are expected to be higher than those using single SNPs in collinear parts of the genome because multiple causal variants will be linked together by the inversion. Thus, inversions offer an opportunity to study the relative importance of additive versus dominance effects in a defined genomic region. We use the inversion genotypes as predictors in association studies with eight morphological traits and compare the contribution of additive and dominance effects on phenotypic variation.

## Results

### Detection and description of inversion polymorphisms in a wild population

#### Linkage disequilibrium patterns

**Fig. 1:**
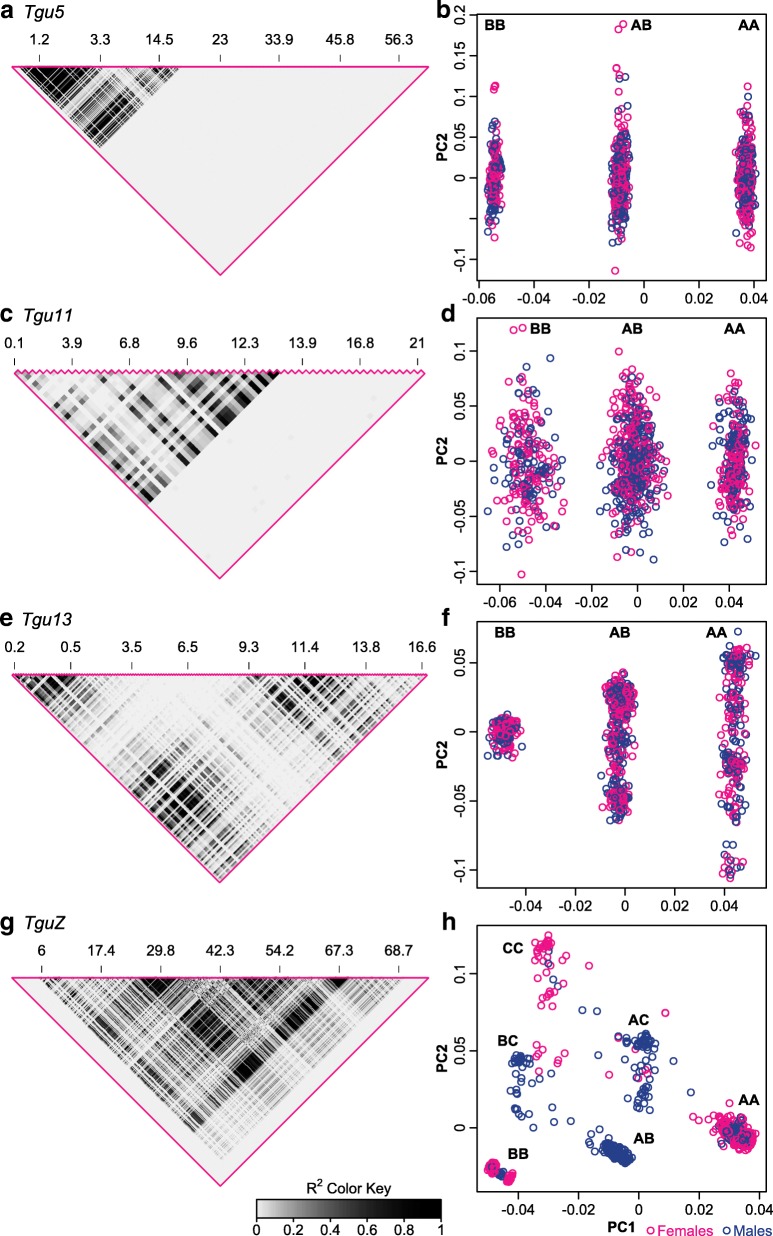
Linkage disequilibrium (LD; *left panel*) and principal component analysis (PCA; *right panel*) results along chromosomes *Tgu5* (**a**, **b**), *Tgu11 *(**c**, **d**), *Tgu13* (**e**, **f**) and *TguZ* (**g**, **h**). In the *right panels*, the letters *A*, *B*, and *C* identify the combination of inversion types (alleles) that individuals (n = 948) carry, with *A* referring to the most frequent and *C* to the least frequent allele. Above the LD plots marker positions in Mb are given. PCA included all SNPs on the respective chromosome. Note that **h** includes a few (n = 18) females that were called as heterozygous for the inversion. These are carriers of occasional double crossovers

In collinear parts of the zebra finch genome, linkage disequilibrium (LD; measured as r^2^) >0.1 extends maximally for 185 kb in our sample of wild Australian birds (Knief U, Schielzeth H, Backström N, Hemmrich-Stanisak G, Wittig M, Franke A, Griffith SC, Ellegren H, Kempenaers B, Forstmeier W: Association mapping of morphological traits in wild and captive zebra finches: reliable within but not between populations, unpublished) (for a representative example, see Additional file 1: Figure S1a). In contrast, four chromosomes (*Tgu5*, *Tgu11*, *Tgu13*, and *TguZ*) showed extraordinarily large linkage blocks, spanning several megabases (12–63 Mb). They showed the typical LD structure of an inversion [53–55], with LD being highest near the presumed inversion breakpoints and lower in the central parts of the inverted region due to gene flux by double crossover (Fig. 1a, c, e, g).

For chromosome *Tgu5* the LD block reaches from 0.96–16.50 Mb, which is equivalent to 25 % of the assembled chromosome and covers 325 genes. The inversion most likely includes the centromere, which is located maximally 5.12 Mb from the proximal chromosome end (Table 1) [56]. On chromosome *Tgu11*, the region of high LD extends from 0.086–12.29 Mb (equivalent to 57 % of the assembled chromosome, spanning 250 genes), covering the most proximal SNP that had been genotyped. The centromere on chromosome *Tgu11* is located at the distal end of the chromosome at around 20 Mb [57] and is thus located outside the LD block (Table 1). On chromosome *Tgu13*, almost the complete assembled chromosome is part of one large LD region (99 %, covering 312 genes), starting from the second proximal SNP and covering the most distal SNP being genotyped (0.15–16.91 Mb). The centromere on chromosome *Tgu13* is located at the distal end of the chromosome [57]. Some parts of the genome assembly are missing at this position [57, 58], yet crossovers between the centromere and a marker located within the LD region occur in heterokaryotypic individuals [57] and we thus conclude that the LD region does not cover the centromere (Table 1). Finally, the physically largest LD block was found on the sex chromosome *TguZ*, extending from 5.91–68.83 Mb, which is equivalent to 86 % of the total chromosome length and covering 619 genes. The centromere on chromosome *TguZ* is located at 27.62–28.12 Mb and is thus included in the LD region (Table 1). Summing up, the inversions on chromosomes *Tgu5* and *TguZ* are pericentric and the ones on chromosomes *Tgu11* and *Tgu13* are paracentric.

**Table 1:** Description of the four large linkage blocks (resulting from inversion polymorphisms) on chromosomes *Tgu5*, *Tgu11*, *Tgu13*, and *TguZ* and the two smaller and less certain ones on chromosomes *Tgu26* and *Tgu27* found in wild Australian zebra finches

Chromosome	Inversion type	SNP ID	Position (bp)	Maximal r^2^	n SNPs
*Tgu5*	Pericentric	WZF00178137	962,370	0.996	152
		WZF00169812	16,503,169	0.184	
*Tgu11*	Paracentric	WZF00035574	86,193	0.187	38
		WZF00031807	12,290,125	0.985	
*Tgu13*	Paracentric	WZF00041237	150,262	0.904	163
		WZF00041448	16,906,706	0.130	
*TguZ*	Pericentric	WZF00231767	5,913,912	0.285	383
		WZF00239958	68,830,532	0.261	
*Tgu26*	Unknown	WZF00114713	657,240	0.101	16
		WZF00114507	2,710,851	0.244	
*Tgu27*	Unknown	WZF00115125	358,632	0.133	23
		WZF00114985	3,097,302	0.553	

Inversions on chromosomes *Tgu5* and *TguZ* had been previously found cytogenetically [45, 48, 50]. Centromere positions were taken from Knief and Forstmeier [57] and Warren et al. [56]. For each chromosome, we list the first and the last SNP (*SNP ID* is our SNP name) that is in LD with the LD region (defined as composite LD r^2^ > 0.1) and we indicate each SNP’s maximal value. *n SNPs* is the number of SNPs genotyped and contributing to the LD region

Weaker signals of long-range LD were also found on chromosomes *Tgu26* and *Tgu27* (Additional file 1: Figure S1c, e), covering 2.05 Mb (42 % of the total chromosome length, covering 57 genes) and 2.74 Mb (59 % of the total chromosome length, covering 166 genes), respectively. Yet, LD patterns were not typical for inversions and principal component analyses (PCAs; see the “Principle component analyses” section below) did not lead to clear clustering (Additional file 1: Figure S1d, f). Hence, we presumed that these are not inversions and did not analyze them further.

We tried to locate the inversion breakpoints with high resolution using the distance and orientation of paired reads from pooled sequencing data. However, we were unable to map any of the breakpoints, suggesting that they are located in genomic regions that are missing in the current genome assembly (for example, in repetitive sequences).

#### Principle component analyses

The four chromosomes found in the LD scan also showed inversion-typical patterns in the PCA (Fig. 1b, d, f, h, principle component loadings: Additional file 1: Figures S2–S5). The three autosomal inversions had two main homozygote haplotype clusters (with the heterozygous individuals in between) and the sex chromosome split into three main homozygote haplotype clusters (with the heterozygous individuals in between). The clusters were well defined on the autosomes but on chromosome *TguZ* the least common haplotype (haplotype C in Fig. 1h) seemed to allow some recombination with each of the two other haplotypes, making the clusters more diffuse. However, both the low average heterozygosity within each cluster of homozygotes compared to heterozygotes (Table 2) and median-joining networks (using Network v4.6.1.1 with standard settings [59]) on phased SNP data at the inversion breakpoint (using Beagle v3.3.2 [60]; Additional file 1: Figure S6) further support the interpretation that the LD regions represent inversion polymorphisms. It should also be noted that chromosomes *Tgu5* and *TguZ* had been previously found cytogenetically to carry pericentric inversions and the breakpoints match precisely to the LD region boundaries [45, 48–50].

**Table 2:** Population genetic descriptive statistics of the four inversion polymorphisms in wild Australian zebra finches

Chromosome	Genotype	Heterozygosity (95 % CI)	Genotype counts		Allele frequency	HWE test
			Males	Females		
*Tgu5*	AA	0.131 (0.0855, 0.178)	154	192	0.595	X^2^_1_ = 1.99, *P* = 0.16
	AB	0.689 (0.644, 0.737)	232	204		Heteroz. deficit
	BB	0.0523 (0.0263, 0.0855)	82	84	0.405	
*Tgu11*	AA	0.0790 (0.000, 0.158)	124	143	0.526	X^2^_1_ = 0.40, *P* = 0.53
	AB	0.493 (0.368, 0.605)	245	218		Heteroz. deficit
	BB	0.214 (0.105, 0.342)	99	119	0.474	
*Tgu13*	AA	0.180 (0.119, 0.240)	129	128	0.525	X^2^_1_ = 0.28, *P* = 0.59
	AB	0.469 (0.411, 0.527)	243	238		Heteroz. excess
	BB	0.170 (0.117, 0.216)	96	114	0.475	
*TguZ* ^a^	AA/AW	0.162 (0.103, 0.230)	140	266	0.596	X^2^_3_ = 4.42, *P* = 0.22
	AB	0.592 (0.521, 0.639)	174	0		Heteroz. excess
	BB/BW	0.0657 (0.0395, 0.0953)	36	155	0.33	
	AC	0.555 (0.496, 0.596)	38	0		
	BC	0.294 (0.265, 0.332)	19	0		
	CC/CW	0.108 (0.0868, 0.143)	4	29	0.074	
	Untyped		57	30		

*Heterozygosity* is the average heterozygosity (across all 152 + 38 + 163 + 383 genotyped SNPs within the inversions) of all individuals within the respective principal component analysis score cluster. Hardy–Weinberg equilibrium (*HWE*) was tested using a chi-square test with the indicated degrees of freedom

^a^Heterozygosity data taken from males only

*CI* confidence interval

From the current analyses we do not know with confidence which arrangement is ancestral and we thus name them according to their allele frequency (A = major haplotype, B = minor haplotype, C = least common haplotype on chromosome *TguZ*; Fig. 1b, d, f, h; Table 2). The major alleles of all four inversion polymorphisms showed remarkably similar frequencies ranging between 0.53 and 0.60 (Table 2). On chromosome *TguZ*, the least common allele (haplotype C) was rare (frequency 0.074; Table 2). All inversion polymorphisms were in Hardy–Weinberg equilibrium (HWE; Table 2) and there was no LD between them, which means that they segregate independently (Additional file 2: Table S1).

#### Pooled heterozygosity and minor allele counts at inversion breakpoints

**Fig. 2:**
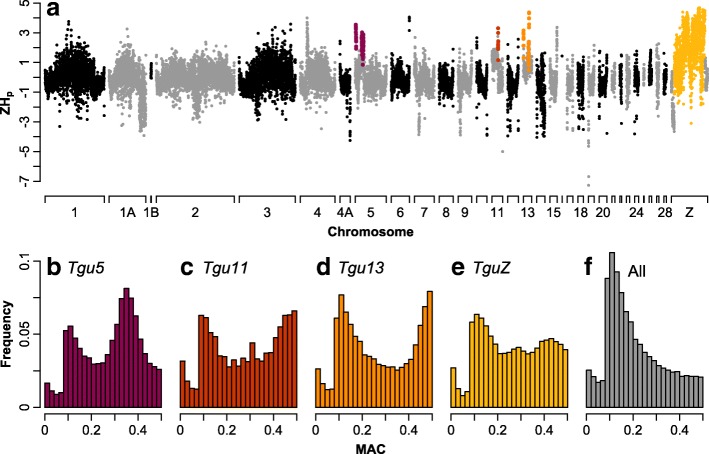
**a** Pooled heterozygosity (ZH_p_) in 50-kb windows along each chromosome in the zebra finch genome. **b**–**e** For the highlighted areas in **a**, which are the presumed inversion breakpoints on the autosomes and the entire inversion interior on the sex chromosome, the minor allele count frequency (*MAC*) spectra are shown for chromosome *Tgu5* with a local maximum at 0.34–0.36 and a frequency of the minor (B) haplotype in the sample of 0.35 (**b**), *Tgu11* with a local maximum at 0.48–0.50 and a frequency of minor (B) haplotype in the sample of 0.47 (**c**), *Tgu13* with a local maximum at 0.48–0.50 and a frequency of minor (B) haplotype in the sample of 0.50 (**d**), and *TguZ* with two local maxima at 0.28–0.30 and 0.42–0.44 and a frequency of the B haplotype in the sample of 0.30 and frequency of the major (A) haplotype in the sample of 0.63 (**e**). **f** For comparison, the MAC of all remaining SNPs peaks at an allele frequency of around 0.1 because SNPs with a lower frequency were not unambiguously called

We calculated pooled heterozygosity (ZH_p_) in 50-kb non-overlapping sliding windows along each chromosome (Fig. 2a). Low values of ZH_p_ are indicative of regions with a high degree of fixation, for example, due to positive selection [61]; high values of ZH_p_ are expected, for example, in regions of local population structure (like inversions) or under balancing selection [62]. We found pronounced peaks in ZH_p_ at the presumed breakpoints of the inversions on chromosomes *Tgu5*, *Tgu11*, and *Tgu13*, whereas ZH_p_ dropped to almost genome-wide average values in the interior of the inversions. Chromosome *Tgu11* had only one such peak, suggesting that the proximal breakpoint is missing in the current genome assembly. Diversity (SNPs per site in a 50-kb window; Additional file 1: Figure S7) was slightly reduced at the presumed breakpoints of every inversion compared to the inversion’s interior (mean SNPs per site ± standard deviation at breakpoints versus interior of 0.017 ± 0.005 versus 0.020 ± 0.005 for *Tgu5*, 0.0057 ± 0.0036 versus 0.018 ± 0.004 for *Tgu11*, and 0.016 ± 0.006 versus 0.022 ± 0.004 for *Tgu13*; 0.021 ± 0.007 collinear autosomal genome-wide average SNPs per site). On chromosome *TguZ*, the entire inversion interior had high ZH_p_ values, which only dropped to the genome-wide average outside the inverted region. Further, diversity on *TguZ* was markedly reduced all along the inverted region, including the presumed breakpoints, and increased to the genome-wide average only outside the inversion (0.0021 ± 0.0015 versus 0.022 ± 0.009, respectively).

The minor allele count frequency (MAC) spectra at the breakpoint regions for all autosomal inversion polymorphisms showed an admixture of the background MAC spectrum (Fig. 2f) with a second MAC distribution whose local maximum matches with remarkable accuracy the allele frequency of the inversion types in those 100 individuals that had been used for the pooled population sequencing (Fig. 2b–e).

### Association analyses and fitness consequences in captive and wild populations

#### Associations with embryo mortality

For each of the inversion polymorphisms, we tested whether heterokaryotypic individuals had higher embryo mortality rates than homozygotes using data from three captive populations of zebra finches. We fitted generalized linear mixed-effects models using embryo mortality as a binomial response variable (0 = embryo survived until hatch, 1 = embryo died naturally; overall proportion of dead embryos, $\overline{\mathrm{x}}\kern0.37em =27.4\ \%,\ \mathrm{n} = 9764$ fertile eggs; for the “Seewiesen” population $\overline{\mathrm{x}}=31.5\ \%,\ \mathrm{n} = 6334$ eggs; for the “Bielefeld” population $\overline{\mathrm{x}} = 22.9\ \%,\ \mathrm{n} = 1170$ eggs; for the “Cracow” population $\overline{\mathrm{x}}=18.4\ \%,\ \mathrm{n} = 2260$ eggs; Table 3) and the inversion genotypes of both parents as two predictors, coded as 1 = heterozygous and 0 = homozygous.

**Fig. 3:**
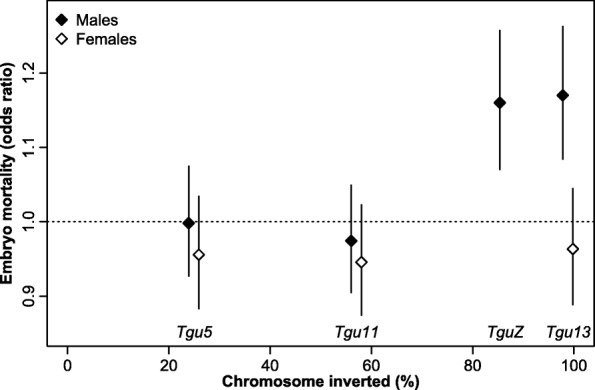
Relationship between the size of an inversion (as percentage of the total chromosome size) and its effect on embryo mortality (meta-analytic summary of dominance effects across three captive populations (“Seewiesen”, “Bielefeld”, and “Cracow”). Shown are the odds ratios ± 1 standard error. An odds ratio >1 indicates an increased rate of embryo mortality in offspring produced by females or males that are heterozygous for one of the four inversions on chromosomes *Tgu5*, *Tgu11*, *Tgu13*, and *TguZ*. The effects on chromosome *Tgu13* and *TguZ* for males both translate into a 4.5 % increase in embryo mortality rate. Only males can be heterozygous for chromosome *TguZ*. For visibility, values on the abscissa were moved 1 % up and down for females and males, respectively

Neither the mother’s nor the father’s inversion genotype had an effect on embryo mortality that survived strict Bonferroni correction (Fig. 3; Additional file 1: Figure S8). Notably, the two inversions which cover almost entire chromosomes (*Tgu13* and *TguZ*) had a weak effect in the expected direction in heterokaryotypic males (meta-analytic odds ratio (OR) [95 % confidence interval] = 1.17 [1.01–1.36] for *Tgu13*, *P* = 0.040, and OR = 1.16 [0.99–1.36], *P* = 0.065 for *TguZ*).

#### Associations with fitness parameters

We fitted generalized linear mixed-effects models using measures of four fitness components as response variables and the inversion genotype simultaneously as an additive (−1 = homozygous for the minor allele, 0 = heterozygous, 1 = homozygous for the major allele, using one degree of freedom) and a dominance (0 = homozygous, 1 = heterozygous) predictor in the three captive populations (“Seewiesen”, “Bielefeld”, and “Cracow”). For females we included fecundity (number of eggs laid) and reproductive success (number of chicks that survived until an age of 35 days). For males we used siring success (total number of eggs sired in communal aviaries) and reproductive success (total number of chicks sired that survived until an age of 35 days). Sample sizes are given in Table 3.

**Table 3:** Sample sizes for the association analyses with embryo mortality and fitness parameters in the three captive zebra finch populations

Population	Parameter	Cage		Aviary	
		Laying	Breeding	Laying	Breeding
Seewiesen	Embryo mortality (number of pairs/number of eggs)	165/930	464/4121	147/765	113/518
	Female fecundity (number of females/number of eggs)	512/10108	327/7539	382/3875	73/655
	Male siring success (number of males/number of eggs)			380/3869	72/655
	Female reproductive success (number of females/number of chicks)		327/1843		73/276
	Male reproductive success (number of males/number of chicks)		305/1843		72/276
Bielefeld	Embryo mortality (number of pairs/number of eggs)				149/1170
	Female fecundity (number of females/number of eggs)				95/1295
	Male siring success (number of males/number of eggs)				95/1295
	Female reproductive success (number of females/number of chicks)				95/556
	Male reproductive success (number of males/number of chicks)				95/556
Cracow	Embryo mortality (number of pairs/number of eggs)	154/1674	52/586		
	Female fecundity (number of females/number of eggs)	22/133	51/776		
	Male siring success (number of males/number of eggs)				
	Female reproductive success (number of females/number of chicks)		51/343		
	Male reproductive success (number of males/number of chicks)		49/343		

**Fig. 4:**
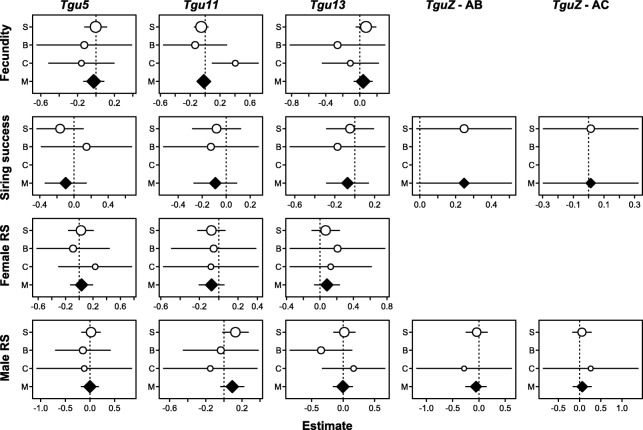
Dominance effects (±95 % confidence intervals) on different fitness parameters (*RS* = reproductive success) in three captive populations (*S* = “Seewiesen”, *B* = “Bielefeld”, *C* = “Cracow” and *M* = meta-analytic summary). Effect sizes are the factor level estimates of square-rooted and Z-transformed fitness components over inversion heterozygosity (while simultaneously fitting additive effects).The point sizes reflect log-transformed sample sizes

Neither in females nor in males did any of the inversion polymorphisms exhibit additive or non-additive fitness effects (Fig. 4; Additional file 1: Figure S9).

#### Frequency-dependent selection

Within the aviary setting we tested whether negative frequency-dependent selection was balancing the inversion polymorphisms (see “Methods”). For this analysis we used two captive populations (“Seewiesen” and “Bielefeld”) and tested whether there was a negative relationship between inversion frequency and fitness. None of the inversion polymorphisms was subject to significant negative frequency-dependent selection. However, the three strongest effects of inversion frequency on fitness parameters were in the expected direction (chromosome *Tgu5* frequency on male reproductive success [nominal *P* = 0.077] and chromosome *Tgu11* frequency on female fecundity [nominal *P* = 0.12] and female reproductive success [nominal *P* = 0.21]; Additional file 1: Figure S10).

#### Segregation distortion

Within three captive populations (“Seewiesen”, “Bielefeld”, and “Cracow”) we tested whether the four inversion polymorphisms were transmitted to the next generation in a fair Mendelian way. None of them showed signs of segregation distortion (Additional file 2: Table S2).

#### Associations with phenotypes

To test whether inversion genotypes had an effect on morphological traits we fitted generalized linear mixed-effects models using eight different Z-transformed phenotypes as response variables (body mass, tarsus length, wing length, beak length, beak depth, beak width, digit ratio, and visible fat deposition scores) and the inversion genotype simultaneously as an additive and a dominance predictor (see above section "Associations with fitness parameters"). For these analyses we used data from three captive zebra finch populations (“Seewiesen”, n = 3233 individuals; “Bielefeld”, n = 1096 individuals; “Cracow”, n = 634 individuals) and from two wild populations (“Fowlers Gap”, n = 939 individuals; “Sydney”, n = 265 individuals).

**Fig. 5:**
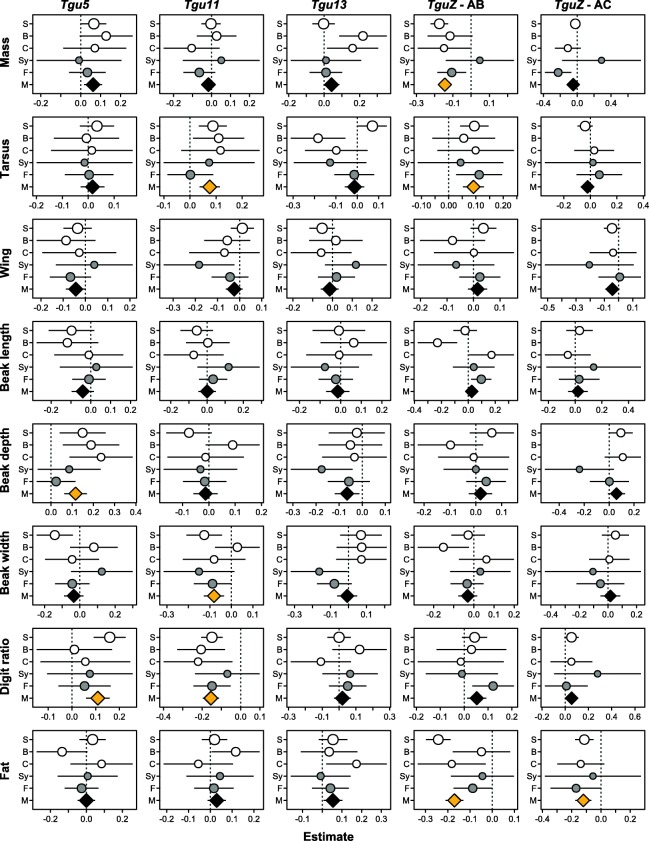
Additive effects of the minor inversion allele ± 95 % confidence intervals on morphological phenotypes across three captive (*white filled circles*; *S* = “Seewiesen”, *B* = “Bielefeld”, *C* = “Cracow”) and two wild zebra finch populations (*grey filled circles*; *Sy* = “Sydney”, *F* = “Fowlers Gap”). *M* = meta-analytic summary (*diamond symbol*; *yellow* if significant after strict Bonferroni correction). Effect size estimates are regression slopes of Z-transformed phenotypes over inversion genotypes (while simultaneously fitting dominance effects) and show the effect of replacing one copy of allele A with allele B (or C in the *rightmost panel*). The point sizes reflect log-transformed sample sizes

The inversions on chromosomes *Tgu5*, *Tgu11*, and *TguZ* had strong additive effects on six out of the eight phenotypes. In total, nine out of 40 associations survived a strict Bonferroni correction (Fig. 5). The major allele A of the inversion on chromosome *TguZ* had the strongest effects overall and increased visible fat deposition (nominal *P* = 1 × 10^−16^) and body mass (nominal *P* = 2 × 10^−14^) and had a negative effect on tarsus length (nominal *P* = 4 × 10^−6^).

None of the inversions exhibited significant dominance effects on any of the phenotypes (Additional file 1: Figure S11).

#### Summary across morphological and fitness phenotypes

**Fig. 6:**
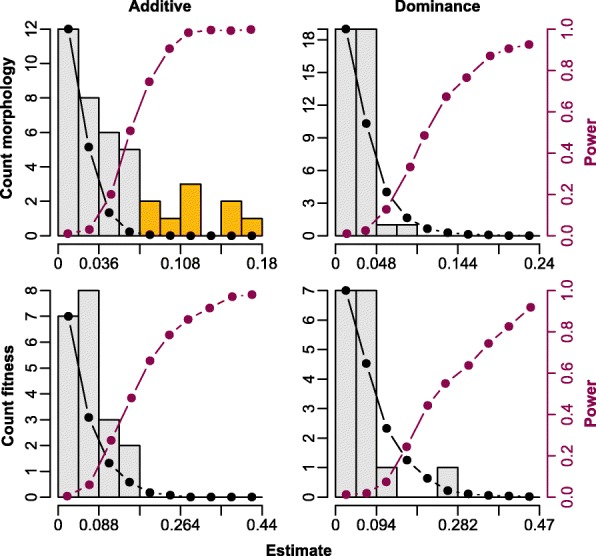
Summary of additive (*left column*) and dominance (*right column*) effect sizes from association studies between inversion genotypes and morphological traits (40 estimates = 8 phenotypes × 5 inversions; *top row*) and of the additive and dominance effect sizes from associations between inversion genotypes and fitness traits (20 [16] estimates = 4 fitness parameters × 5 inversions [minus 4 *TguZ* dominance effects in females]; *bottom row*). Empirical effect sizes are shown as the *light grey bars* overlaid with the null distribution as a *black line*. Effects that survived strict Bonferroni correction are highlighted in *yellow*. Power for a given effect size is overlaid in *purple* with its corresponding *y-axis* on the right. We estimated the null distribution (and the power values) by permuting the inversion genotypes within sexes (and adding/subtracting the corresponding effect sizes to/from the phenotypic values) and fitting the same mixed models as for the empirical data set (see “Methods” for details). For illustration, the null distribution was scaled to overlap the first bar in the histogram of the empirical estimates completely. Partial regression coefficients of additive and dominance effects are not directly comparable the way we standardized and fitted them and thus their null distributions differ (dominance effects reach higher values than additive effects because their variance is smaller; see also [114, 115])

The effects of inversion genotypes on morphology and fitness could be so small that they were not detectable in our association studies due to low power (thereby committing a type II error; Fig. 6). This is especially true for the fitness components because sample sizes were smaller and effect sizes are expected to be smaller, at least for the additive genetic component (since natural selection should reduce the amount of additive genetic variance in fitness [63]). To obtain the expected distribution of effect sizes under randomness (null distribution) and to estimate the power for different effect sizes we used a permutation approach (see “Methods” for details).

The 40 empirical additive effect size estimates on morphology (5 comparisons between inversions × 8 phenotypes) clearly exceeded the random expectation (observed mean ± standard error (SE) = 0.0494 ± 0.0069, expected mean ± SE = 0.0163 ± 0.0002), suggesting that several of the associations which did not survive a strict Bonferroni correction were in fact real (Fig. 6). In line with this, power to detect small additive effect sizes was relatively low. In contrast, the 40 empirical dominance effects on morphology did not deviate from the random expectation (Fig. 6). In fact, the mean of the 40 dominance effects (0.0251 ± 0.0029) was slightly smaller than the mean of the random expectation (0.0300 ± 0.0004) and power to detect large effects was high. Similarly, neither the 20 empirical additive effect size estimates on fitness (5 comparisons between inversions × 4 fitness components) nor the 16 dominance effects (the same 20 associations excluding dominance effects on female fitness components on chromosome *TguZ*) deviated from the random expectation, despite high power for detecting large effect sizes (Fig. 6). Because we specifically expected to find positive dominance effects (heterosis) for fitness, we calculated the overall mean of the 16 dominance effects and found an effect size that was very close to zero (weighted d = 0.0019, *P* = 0.91).

## Discussion

Here we describe four large inversion polymorphisms (12–63 Mb) in wild Australian zebra finches using molecular and population genetic tools. Two of them had been identified previously in cytogenetic screens because they shift the position of the centromere [45, 48–50]. In total, the inverted regions span at least 8.7 % of the zebra finch genome and 8.1 % of all annotated genes (based on the Ensembl80 gene predictions). Although all polymorphisms are in HWE, their remarkably similar allele frequencies (range of the major allele 0.53–0.60) may indicate some sort of balancing selection acting on them. We find tentative evidence that the largest two inversions increase embryo mortality in heterokaryotypic males (but not in females), which makes their high allele frequencies even less likely to be due to drift alone. However, although the inversions have an additive effect on several morphological traits, we do not find any dominant gene action and no balancing effects on several aspects of fitness in three captive zebra finch populations.

### Ruling out other processes leading to high LD

In this study we used PCAs and LD patterns to detect inversions and to genotype the wild-caught individuals [64–68]. LD in the four inversions was increased 26–137 times compared to same-sized regions in the rest of the genome, which is still an underestimate since in the collinear parts of the genome, LD estimates arise from sampling noise alone (we use r^2^ to measure the strength of LD which can never be negative and never reach 0) [69]. We further backed-up our interpretation by looking at population genomic patterns, which are typical for inversion polymorphisms with the highest LD values at and between the inversion breakpoints (see also the “Detection and description of inversion polymorphisms in a wild population” section in the “Results”) [8, 53, 55]. In addition, for two of the chromosomes (macrochromosomes *Tgu5* and *TguZ* with pericentric inversions) there is independent cytogenetic evidence for polymorphic inversions [45, 48–50] (see below for details).

Regions of high LD in the genome can arise by processes other than inversions that suppress recombination; demographic events (inbreeding, admixture, bottlenecks), genetic drift in regions with low recombination, or natural selection are such mechanisms [70]. The wild Australian zebra finch population has been increasing to a current effective population size of 1.3 to 7 million [52] with no traces of inbreeding [71], admixture, bottlenecks, or population structure [51, 52]. Hence, demographic events cannot have caused the observed high LD on just four chromosomes while maintaining very low LD values on all other chromosomes. We can also reject genetic drift as a mechanism creating the observed high LD spanning more than 12 Mb. In zebra finches, recombination is highly biased towards the telomeres with large “recombination deserts” in the center of the macrochromosomes [58, 72]. Chromosome *Tgu2* is the largest chromosome in the zebra finch genome with the largest “recombination desert” (and the lowest recombination rate [58]). However, there is no such long-range LD on chromosome *Tgu2* (Additional file 1: Figure S1a) and neither on any other chromosome. There is a sign of drift in a region with low recombination visible on chromosome *Tgu2*, namely at the centromere between 81 and 82 Mb, thus spanning less than 1 Mb (Additional file 1: Figure S1a). So, could selection in the absence of inversions have caused the regions of high LD we observed? Increased LD due to selection is usually transient [73] and decays rapidly after a beneficial allele has been fixed [74]. In the face of substantial recombination, strong and ongoing (epistatic) selection would be required to keep regions larger than 12 Mb in high LD [70]. However, we did not find any evidence for a selective advantage. Another typical sign of selection is reduced nucleotide diversity [73], but this was only visible on chromosome *TguZ* (Additional file 1: Figure S7). Moreover, the abrupt increase in diversity at the presumed breakpoints on chromosome *TguZ* is more likely the result of selection acting on an inversion polymorphism [75]. Taken together, the most likely explanation for the observed long-range LD on chromosomes *Tgu5*, *Tgu11*, *Tgu13*, and *TguZ* is that they harbor polymorphic inversions.

### Comparison to cytogenetic results

Previous cytogenetic analyses detected pericentric inversions on chromosomes *Tgu5* and *TguZ* [45, 48–50]. In these analyses, the inversion on chromosome *Tgu5* covered around 31 % of the chromosome with the proximal breakpoint located close to the telomere of the short arm [49], which matches well with our LD region (LD block reaches from 0.96–16.50 Mb, corresponding to 25 % of the chromosome length). On chromosome *TguZ*, cytogenetic analyses located the proximal inversion breakpoint at around 5.85 Mb (but in a highly repetitive region which is missing in the current genome assembly) and the distal breakpoint beyond 65.38 Mb [50]. Again, this matches well with our LD region (LD block reaches from 5.91–68.83 Mb). LD and PCA patterns for chromosomes *Tgu11* and *Tgu13* are very much the same as for chromosomes *Tgu5* and *TguZ*. The inversions on these two microchromosomes most likely have not been discovered in the cytogenetic studies because these chromosomes cannot be identified unambiguously and are usually disregarded in such studies. Furthermore, the inversion on chromosome *Tgu11* is paracentric and thus does not shift the position of the centromere and on chromosome *Tgu13* the change of the centromere position is most likely invisible.

### Inversion polymorphisms on chromosomes *Tgu5* and *Tgu11*—smaller and less complex

On chromosome *Tgu5*, the inversion is pericentric and on chromosome *Tgu11* paracentric (see also [45, 48, 57]). Compared to the inversions on chromosomes *Tgu13* and *TguZ* (described further in the next section), the inverted segments on chromosomes *Tgu5* and *Tgu11* span smaller proportions of the corresponding total chromosome lengths (covering 25 % and 57 % of the total chromosome, respectively). In the PCAs, individuals were only separated along PC1. Individual scores on PC2 showed a normal distribution, which indicates that there is no additional population substructure [76, 77] due to a second rearrangement included in, overlapping with, or independent of the first. In that sense, the inversions on chromosomes *Tgu5* and *Tgu11* are less complex than those on chromosomes *Tgu13* and *TguZ* (see the next section). Individual SNPs loaded only on PC1 with the strongest loadings close to the breakpoints. Accordingly, the median-joining networks formed only two separated haplotype clusters and also the LD patterns suggested that these are simple single inversions. LD and pooled heterozygosity were highest at the presumed breakpoints and dropped to the central regions, which are typical signs of gene flux due to double crossovers between two simple arrangements [8]. Parsimoniously, if double crossovers do occur, we would also expect that single detrimental crossovers should occur occasionally between the arrangements, leading to unbalanced gametes and embryo mortality, but we did not observe any increased embryo mortality rate in heterokaryotypic individuals.

At least one single crossover per chromosome is needed to ensure the proper segregation of homologous chromosomes in meiosis [78]. On chromosomes *Tgu5* and *Tgu11*, however, the collinear parts of the chromosome are large and a crossover is likely to be initiated there, with no adverse effect on the meiotic products [1]. Alternatively, the inversions may be too rigid to synapse (a loop structure needs to be formed within the inverted region), thereby suppressing recombination [5]. We suspect that detrimental single crossovers within the inverted region in heterokaryotypic individuals happen so rarely that they fall below the detection limit in our analysis of embryo mortality. In line with this interpretation, a recent cytogenetic study on the inversion on chromosome *Tgu5* did report no loop formation and no crossovers in 230 meioses from three heterokaryotypic individuals (including females and males) [49]. The absence of an inversion loop suggests that the homologs synapse heterologously [49].

On chromosome *Tgu5*, heterozygosity (Table 2) and diversity (the spread in the median-joining network) within cluster B is low, suggesting that it increased in frequency in the population and that type A is the ancestral state. Using the same line of argument, on chromosome *Tgu11*, type A spread in the population and type B is the ancestral state.

### Inversion polymorphisms on chromosomes *Tgu13* and *TguZ*—complex and costly

The inversion on chromosome *Tgu13* is paracentric and at least one inversion on chromosome *TguZ* is pericentric (see also [50]). The inverted regions on both chromosomes are very large relative to the corresponding total chromosome lengths (covering 99 % and 86 % of the total chromosome, respectively, but note that the assembly of chromosome *Tgu13* appears to be incomplete). Our PCA for chromosome *TguZ* showed at least three large haplotype clusters (A, B, and C), but also the higher principle component PC3 deviated from a normal distribution, suggesting an even more complex situation and an independent linkage block at the distal inversion breakpoint (Additional file 1: Figure S5). In line with this interpretation, individual SNPs loaded in complex patterns on PC1 to PC3 and the outermost breakpoints were not particularly defined. Itoh et al. [50] described a single large pericentric inversion on chromosome *TguZ* and we suppose that they identified the two most common types A versus B (B together with C) because our breakpoint locations match with the data in Itoh et al. [50] and there is apparently no LD between types B and C in the region where their tagging length polymorphism (deletion/insertion) is located (Additional file 1: Figure S12). However, in that case the allele frequency estimates by Itoh et al. [50] in wild Australian zebra finches deviate from ours (sums across all sampling locations, assuming that the common type is allele A: A = 61, B + C = 9; for our sample A = 758, B + C = 514, Fisher’s exact test *P* = 8 × 10^−6^). This could be explained if their tagging marker is not reliably linked to the inversion in the wild (the marker is only accurate in 28 out of the examined 30 cases in two captive populations). Note that we used a total of six tagging SNPs that showed perfect clustering in 948 wild birds.

The median-joining network and the number of shared SNPs suggest that haplotypes B and C on chromosome *TguZ* are more closely related with each other than with haplotype A. Judging from the fuzzy clusters formed in the PCA, gene flux between arrangements seems to happen, either between haplotypes A and C or between haplotypes B and C or between both of the pairs. Thus, inversion types B and C could be more related because of their shared ancestry or because of gene flux and in the end we cannot separate these two possibilities.

The PCA on chromosome *Tgu13* separated individuals largely along PC1. However, PC2 distinguished between at least two groups within inversion type A; yet these groups were not completely separated, indicating some more extensive gene flux between them. The higher principle components (≥PC3) were normally distributed, suggesting that there is no additional population substructure [76, 77]. The LD patterns on chromosome *Tgu13* suggest that there is gene flux between the two main arrangements (types A and B) due to double crossovers [8] (Fig. 1).

There is tentative evidence that mortality rates are increased in embryos sired by heterokaryotypic males for both chromosomes *Tgu13* and *TguZ* (by a weighted average of 4.5 % for each of the chromosomes across populations). We suspect that these effects are not type I errors resulting from multiple testing because also in human males noticeable rates of unbalanced gametes are produced only when an inversion (both para- and pericentric) spans more than half of the chromosome [5, 11, 12]. In fact, our Fig. 3 shows a remarkable similarity to Fig. 6b in Anton et al. [5]. However, assuming that the rate of unbalanced gametes translates directly into embryo mortality, the effect in humans is an order of magnitude (12-fold) larger than in zebra finches [5], indicating that zebra finches evolved a rather effective way to decrease recombination within inversion heterokaryotypes. Interestingly, the median-joining networks and PCA results suggest that there is a succession of inversions on chromosomes *Tgu13* (within haplotype A) and *TguZ* (haplotypes B and C appear to be more closely related), and accumulating inversions on a chromosome may be a way to increasingly suppress recombination between inversion haplotypes (as, for example, in the *t*-complex in mice [40]).

Heterokaryotypic female zebra finches did not show increased rates of embryo mortality for the inversion on chromosome *Tgu13*, which suggests that they are either able to pass on abnormal meiotic products to the second polar body (as found in *Drosophila* and maize in case of paracentric inversions [6, 14]) or that they are able to shut down recombination within the inverted region almost completely (as suggested for pericentric inversions in grasshoppers [1]).

For both chromosome *Tgu13* and *TguZ* it is difficult to conclude which haplotypes represent the ancestral states from patterns of diversity or the median-joining networks. Within the inversion on chromosome *TguZ*, SNPs per site were reduced tenfold compared to the collinear outer parts of *TguZ* (Additional file 1: Figure S7; which was also found by Balakrishnan and Edwards [52]). Hence, the patterns of diversity indicate that all three haplotypes (A, B, and C) are rather recently derived, having replaced the high ancestral diversity that is still present on the distal ends of chromosome *TguZ* (Additional file 1: Figure S7). However, the minimal sojourn time of at least one of the rearrangements is supposed to be at least 1.2–2.8 million years, which is the estimated split time, with little subsequent gene flow, between Timor and Australian zebra finches [52]. Supposedly, both subspecies are polymorphic for one of the rearrangements [50].

### Fitness effects: no heterosis for viability in the wild or for fitness in captivity

The inversions on chromosomes *Tgu13* and *TguZ*, and probably also to a lesser extent the ones on chromosomes *Tgu5* and *Tgu11*, should be costly in terms of increased embryo mortality whenever they are in the heterozygous state in males. Given an effective population size of wild Australian zebra finches of 1.3 × 10^6^ to 7 × 10^6^ [52], it is unlikely that the polymorphisms would have escaped purifying selection and be at frequencies (0.53–0.60) close to their fitness minimum (at an allele frequency of 0.5 the maximal number of individuals are heterozygous), if they do not confer a fitness advantage to their carriers.

The simplest condition for a balanced polymorphism with two alleles is given when both homozygotes have lower fitness than heterokaryotypic individuals (heterosis [1]). In our sample of wild zebra finches, all four inversion polymorphisms were in HWE, indicating that there was no heterosis for viability at the time of sampling the individuals. However, it is possible that heterosis is only expressed during stressful environmental conditions in the wild, such as during a severe drought. Such selective events could be so rare that they did not happen during the few years in which the sampled individuals lived. Furthermore, deviations from HWE are not necessarily expected if heterotic superiority depends on fecundity or siring success rather than viability [1]. Thus, we tested whether the inversions showed heterotic superiority with respect to several other aspects of fitness (female fecundity, male siring success, and the number of offspring produced) in three captive populations of zebra finches. The average of all effect sizes was close to zero (weighted d = 0.0019, *P* = 0.91), suggesting that none of the four inversions exhibits heterosis, at least in a captive environment. The number of offspring produced is not independent of embryo mortality and the observation of no underdominance for fitness either means that the reduction in fitness is compensated for or that we lack the power to detect it, given that the effect on embryo mortality was barely significant using almost 10,000 eggs.

Heterotic superiority could be due to direct overdominance (resulting from either the inversion breakpoints themselves or an allele fixed on both haplotypes and conferring a heterozygote advantage) or due to recessive deleterious mutations captured by or accumulating on the inverted haplotype (“associative overdominance”) [23]. If indeed heterotic superiority was stabilizing the inversions, we should be able to detect it also in captivity, judging from the strong inbreeding effects that have been observed in the captive Seewiesen population on morphology and fitness components [79, 80]. Thus, although we cannot rule out heterosis completely, other forms of balancing selection are more likely to keep the inversion polymorphic, which do not require heterokaryotypic superiority and do not lead to deviations from HWE, such as (negative) frequency-dependent selection [1], meaning that individuals carrying the rare inversion type have higher fitness than those carrying the more common type. In several *Drosophila* species, negative frequency-dependent selection stabilized inversion polymorphisms [33, 34, 81]: after the inversion frequencies were experimentally perturbed in a population, individuals with the rare inversion type had higher fitness and the inversion frequencies quickly returned to their equilibrium values in subsequent generations. Interestingly, negative frequency-dependent selection can theoretically lead to stable equilibria even if inversion polymorphisms are underdominant [82]. However, we did not find any significant evidence for frequency-dependent selection in the captive environment. Yet, the strongest effects of inversion frequency on fitness parameters were in the direction expected under negative frequency-dependent selection and this might deserve further study.

### Morphological effects: all additive, no dominant gene action

We found remarkable additive genetic effects of the inversion genotypes on several morphological traits, which were highly consistent across populations (Fig. 5). Only one test for heterogeneity between populations was significant after Bonferroni correction, which was the association between beak length and haplotype A versus B on chromosome *TguZ* (Cochran’s Q test *P* = 0.026 [83]). In contrast to the strong additive effects on morphology, none of the four inversion polymorphisms exhibited dominant gene action.

Additive genotype–phenotype association studies typically find small effects of individual SNPs on a phenotype [84, 85] and associations are often difficult to replicate between populations due to differences in LD structure [86]. Recently, we tested several promising causal SNPs in collinear parts of the zebra finch genome for an additive association with the same morphological phenotypes and in the same populations as the ones studied here. Individual SNP effects were small and not consistent across populations (Knief et al., unpublished). The contrast between the diminishingly little additive effects of individual SNPs in collinear parts of the genome and the large and consistent additive effects of inversions is most likely due to differences in LD and highlights the polygenic nature of the quantitative traits under study. Whereas in collinear parts of the zebra finch genome LD decays rapidly [52], making associations hard to detect, inversions capture hundreds of alleles in extended defined haplotypes, which do not or hardly ever recombine. Thereby, they combine the additive effects of many causal alleles.

## Conclusions

Large inversion polymorphisms are abundant in the estrildid finch family [45–47]. Here we describe inversion polymorphisms in one species belonging to this family, the zebra finch. We find polymorphic inversions on at least four out of its 32 annotated chromosomes. In each case, a novel haplotype has spread to about 50 % allele frequency and has persisted for an extended period of time. However, exactly when and in which species these inversions arose remains to be worked out. It will be interesting to see whether the polymorphisms are shared between species (as suggested in juncos [42]) and if so whether they introgressed into zebra finches (as it is apparently the case in white-throated sparrows [22]). Of course, we do not have any information on the ancestral fitness landscape of these inversion polymorphisms and thus on the mechanisms that led to their establishment, but we tested which selective forces maintain them in a polymorphic state at present. However, these forces remain elusive: we found no signs of heterosis for viability in a wild population or for other fitness-related traits in captivity despite our ability to measure fitness effects of recessive deleterious mutations in captivity [80]. Nevertheless, some benefit to the individual (undetected heterosis or frequency-dependent selection) or to the genotype itself (segregation distortion [87]) is expected, since a small cost remains: heterokaryotypic males produce a higher proportion of inviable embryos, presumably due to single crossovers within the inverted region. It appears that past selection has effectively minimized this cost: (1) “Small” inversions (chromosomes *Tgu5* and *Tgu11*) do not observably increase the proportion of inviable embryos produced by heterokaryotypic individuals. Perhaps these inversions do not synapse regularly in meiosis, thereby reducing the risk of detrimental crossovers. (2) Heterokaryotypic females do not exhibit increased rates of embryo mortality even for the largest inversion on chromosome *Tgu13.* Thus, they may have found a way to deposit the abnormal meiotic products (the dicentric single-crossover chromatids in case of a paracentric inversion) to the polar bodies. (3) The effects on embryo mortality in heterokaryotypic males for the two largest inversions on chromosomes *Tgu13* and *TguZ* are an order of magnitude smaller than those reported in humans. We suspect that this could be due to selection favoring repeated inversions on the same chromosome, thereby effectively suppressing pairing of the inversion types during meiosis and inhibiting detrimental crossovers. Additionally, the highly skewed distribution of recombination events towards the chromosome ends in zebra finches [58] and other Estrildidae species [72] may minimize crossovers in the inverted regions. The interior parts of zebra finch chromosomes show large recombination deserts (15-fold lower recombination rate compared to the chicken [58]) and it is possible that the underlying molecular mechanism was favored by selection because it also suppresses recombination in inverted regions. Testing this idea quantitatively would require an improved assembly of chromosomes *Tgu11* and *Tgu13*.

## Methods

### Inversion discovery in wild zebra finches

#### Study population and phenotypes

We took blood samples from 1059 wild adult zebra finches (530 females, 529 males) at Fowlers Gap, NSW, Australia, in two places (S 30°57’ E 141°46’ and S 31°04’ E 141°50’) from October to December 2010 and in April/May 2011. A detailed description of the study sites and catching procedure using a walk-in trap at feeders is provided in Griffith et al. [88] and Mariette and Griffith [89]. In this study we refer to this population as “Fowlers Gap”.

The following phenotypes were measured on all birds: right tarsus length, right wing length, beak length, beak depth, beak width, ratio of the length of the second to fourth digit of the right foot (measured twice and averaged), and body mass. Further details on the measurement procedures and summary statistics are given in Knief et al. (unpublished, available upon request). We included a score-based measure of visible fat on the ventral side at the furcular depression and at the abdomen [90].

#### Population-level SNP data and sequencing

We sequenced pooled non-barcoded DNA samples from 100 of the 1059 “Fowlers Gap” individuals on the Illumina HiSeq 2000 platform (paired-end) at the Institute of Clinical Molecular Biology (IKMB) at Kiel University, Germany. Software input parameters are provided in Knief et al. [71]. Briefly, after mapping reads to the zebra finch genome assembly (WUSTL 3.2.4 [56]) using bwa (v0.5.9 [91]), we calculated an average genome coverage of 247.5× (using BEDTools v2.17.0 [92]) and called around 23 million SNPs using GATK (v2.1-11-g13c0244 [93]). SNPs with a minor allele count frequency (MAC) below 0.1 were rarer than expected due to an ascertainment bias in the SNP discovery pipeline [71].

Pooled population sequencing allows estimating diversity and allele frequencies across the genome [94]. Although individual-based data were missing, we calculated a measure of heterozygosity (pooled heterozygosity, H_p_) in 50-kb non-overlapping sliding windows along the autosomes [61] as H_p_ = 2 × ∑n_MAJ_ × ∑n_MIN_/(∑n_MAJ_ + ∑n_MIN_)^2^, where n_MAJ_ and n_MIN_ are counts of reads covering the major and minor allele, respectively, and ∑n_MAJ_ and ∑n_MIN_ are the sum of all these counts in a 50-kb window. We transformed the H_p_ values into Z-scores (ZH_p_) as ZH_p_ = (H_p_ − μH_p_)/σH_p_.

In order to locate the inversion breakpoints with high resolution, we used the BreakDancer (v1.1) [95] and “clipping reveals structure” (CREST v0.0.1) [96] algorithms with default settings on our mapped paired-end pooled-sequencing reads. BreakDancer makes use of read pairs which are separated by unexpectedly large distances or which are oriented in a parallel manner in comparison to the reference genome to identify structural variants. On the other hand, CREST uses the unaligned portion of a sequencing read (soft-clipping information stored along with the mapped reads) and maps it to the reference genome to predict structural variants.

#### SNP chip design

From the 23 million SNPs we designed an Illumina Infinium iSelect HD Custom BeadChip with 6000 attempted bead types [71]. In short, 884 SNPs resided within candidate genes for an association study and were not used for the present study and 4405 SNPs covered all assembled chromosomes except chromosome *Tgu16*. We attempted to position at least 40 physically evenly spaced SNPs on each chromosome, yet this was not possible for chromosomes *Tgu1B* (n = 33 SNPs) and *Tgu25* (n = 24 SNPs) because too few SNPs passed our filtering procedure [71]. In regions of the genome where the pooled heterozygosity was exceptionally high we increased the SNP density. Overall we intended to genotype 5289 SNPs (which summed up to 6000 bead types because we did not exclude C/G and A/T SNPs that require two bead types for genotyping) and the final chip delivered by Illumina contained 4553 of these SNPs, with drop-outs being randomly distributed along chromosomes (Knief et al., unpublished).

Median marker spacing of SNPs on the chip was 243.17 kb (interquartile range [IQR] = 16.68–343.70 kb) on macrochromosomes (chromosomes *Tgu1*–*Tgu5*, *Tgu1A*), 239.03 kb (IQR = 20.57–355.14 kb) on microchromosomes (all other autosomes) and 174.63 kb (IQR = 161.11–179.40 kb) on chromosome *TguZ*.

#### Individual genotyping and quality control

All 1059 “Fowlers Gap” individuals were genotyped for the 4553 SNPs at the IKMB at Kiel University. Quality control was done using the R package GWASTools (v1.6.2) [97] and details are provided in Knief et al. [71]. In summary, we removed 111 individuals with a missing call rate larger than 0.05 (which was due to DNA extraction problems, but these birds were genotyped in the follow-up study; see the “Follow-up genotyping and phenotyping in captive populations” section below), leaving 948 individuals. Further, we removed 152 SNPs that did not form defined genotype clusters, or had high missing call rates (missing rate >0.1), or were monomorphic, or deviated strongly from HWE (Fisher’s exact test *P* < 0.05/4553), or because their position in the zebra finch genome assembly was likely not correct, leaving 4401 SNPs.

#### LD calculations

Inversion polymorphisms lead to extensive LD across the inverted region, with the highest LD near the inversion breakpoints because recombination in these regions is almost completely suppressed in inversion heterozygotes [53–55]. To screen for inversion polymorphisms we did not resolve genotypic data into haplotypes and thus based all LD calculation on composite LD [98]. We calculated the squared Pearson’s correlation coefficient (r^2^) as a standardized measure of LD between every two SNPs on a chromosome genotyped in the 948 individuals [99, 100]. In order to calculate and test for LD between inversions we used the methods described in [101] to obtain r^2^ and *P* values for loci with multiple alleles.

#### Principle component analyses

Inversion polymorphisms appear as a localized population substructure within a genome because the two inversion haplotypes do not or only rarely recombine [66, 67]; this substructure can be made visible by PCA [102]. In case of an inversion polymorphism, we expected three clusters that spread along principle component 1 (PC1): the two inversion homozygotes at both sides and the heterozygotes in between. Subsequently, the principal component scores allowed us to classify every individual as being either homozygous for one or the other inversion genotype or as being heterozygous [67].

We performed PCA on the quality-checked SNP set of the 948 individuals using the R package SNPRelate (v0.9.14) [103]. On the macrochromosomes, we first used a sliding window approach analyzing 50 SNPs at a time, moving five SNPs to the next window. Because the sliding window approach did not provide more details than including all SNPs on a chromosome at once in the PCA, we only present the results from the full SNP set per chromosome. On the microchromosomes, the number of SNPs was restricted and thus we only performed PCA including all SNPs residing on a chromosome.

In collinear parts of the genome composite LD >0.1 does not extend beyond 185 kb (Additional file 1: Figure S1a; Knief et al., unpublished). Thus, we also filtered the SNP set to include only SNPs in the PCA that were spaced by more than 185 kb (filtering was done using the “earliest finish time” greedy algorithm [104]). Both the full and the filtered SNP sets gave qualitatively the same results and hence we only present results based on the full SNP set, also because tag SNPs (see the “Tag SNP selection” below) were defined on these data. We present PCA plots based on the filtered SNP set in Additional file 1: Figure S13.

#### Tag SNP selection

For each of the identified inversion polymorphisms we selected combinations of SNPs that uniquely identified the inversion types (composite LD of individual SNPs r^2^ > 0.9). For each inversion polymorphism we calculated standardized composite LD between the eigenvector of PC1 (and PC2 in case of three inversion types) and the SNPs on the respective chromosome as the squared Pearson’s correlation coefficient. Then, for each chromosome, we selected SNPs that tagged the inversion haplotypes uniquely. We tried to pick tag SNPs in both breakpoint regions of an inversion, spanning the largest physical distance possible (Additional file 2: Table S3). Using only information from the tag SNPs and a lenient majority vote decision rule (i.e., the majority of the tag SNPs determines the inversion type of an individual, missing data are allowed), all individuals from Fowlers Gap were assigned to the correct inversion genotypes for chromosomes *Tgu5*, *Tgu11*, and *Tgu13* (Additional file 1: Figure S14a–c). Since clusters are not as well defined for chromosome *TguZ* as for the other three autosomes, there is some ambiguity in cluster borders. Using a more strict unanimity decision rule (i.e., all tag SNPs must specify the same type, missing data are not allowed), the inferred inversion genotypes from the tag SNPs correspond perfectly to the PCA results but leave some individuals uncalled (Additional file 1: Figure S14d).

### Follow-up genotyping and phenotyping in captive populations

#### Study populations

To study phenotypic and fitness effects of the inversion polymorphisms we genotyped all 15 tag SNPs (Additional file 2: Table S3) in an additional 5229 birds stemming from four different populations: (1) a captive population held at the Max Planck Institute for Ornithology in Seewiesen, Germany (n = 3233 individuals; study population 18 in Forstmeier et al. [51]) with a complete pedigree covering eight generations, of which the last seven were genotyped for the tag SNPs completely. In the following, we refer to this population as “Seewiesen”. (2) A recently wild-derived population held at the Max Planck Institute for Ornithology in Seewiesen (n = 1096 individuals; originating from study population 4 in Forstmeier et al. [51]) with a complete pedigree covering six generations, of which the last four generations were genotyped completely. We refer to this population as “Bielefeld”. (3) A population that was produced by crossing individuals from a captive population held in Cracow (study population 11 in Forstmeier et al. [51]) with the Seewiesen population (n = 634 individuals) with a complete pedigree covering three generations, of which all generations were genotyped completely. We refer to this hybrid population as “Cracow”. (4) Wild birds that were caught at Fowlers Gap and Sturt National Park, NSW, and then held at Macquarie University in Sydney, Australia, and additional wild birds from Fowlers Gap, which were excluded in the initial genotyping due to DNA extraction problems (see above; n = 265 individuals without pedigree information). We refer to these birds as “Sydney”.

#### Genotyping, quality control, and inversion haplotype inference

Tag SNPs (n = 15, chromosomes *Tgu5* + *Tgu11* + *Tgu13* + *TguZ* = 3 + 3 + 3 + 6) were included in three Sequenom genotyping assays (plexes), which in total consisted of 62 SNPs. All 5229 individuals were genotyped according to the manufacturer’s users guide on the Sequenom MassARRAY iPLEX platform [105] at the IKMB at Kiel University. Genotypes were called using the MassARRAY Typer (v4.0) software with standard settings.

The quality control procedure of genotype calls has been described previously and involved inheritance checks using PedCheck (v1.00) [106], the inference of null alleles, and a comparison of 16,013 genotype calls of individuals that were genotyped using both the Illumina and Sequenom genotyping platforms. All tests indicated high genotyping accuracy.

We inferred inversion genotypes for each individual as in the “Fowlers Gap” population using a majority vote decision rule. Founders of all four populations that produced offspring (n = 239 individuals) were run on both the Illumina and Sequenom genotyping platforms. Thus, we used the SNP loadings on PC1 and PC2 from the PCA of the Fowlers Gap birds on the population founders to calculate a PCA score for each individual (Additional file 1: Figure S15a–d) and compared the inversion genotypes inferred by PCA and tag SNPs. There was complete agreement between the two methods for the autosomal inversion genotypes (Additional file 2: Table S4). In the Bielefeld population a recombinant haplotype for chromosome *TguZ* was common (26 out of 74 founder individuals; Additional file 1: Figure S15d) and we changed the majority vote decision rule to a unanimity decision rule, which reduced the number of individuals assigned to a specific inversion genotype and removed all wrongly assigned individuals (Additional file 1: Figure S15d, Additional file 2: Table S4). Previously, 1062 Seewiesen individuals had been genotyped with a different set of 37 SNPs on chromosome *TguZ* [58] and we compared the inversion haplotype inference between Sequenom and the PCA results using these 37 SNPs and they agreed completely (Additional file 2: Table S4). Using these inference rules there was not a single inheritance error of an inversion genotype out of 35,584 inheritance events. Inversion allele frequencies in the four follow-up populations are provided in Additional file 2: Table S5.

#### Morphological phenotyping

The same morphological phenotypes were measured for every bird using the same methodology as for the wild birds from the “Fowlers Gap” population. Each phenotypic measurement was taken once (twice for digit ratio) per individual such that phenotypic values and their measurement errors between the “Fowlers Gap” and the other populations are comparable [39]. Descriptive statistics for each trait (except visible fat deposition) are summarized in Knief et al. (unpublished, available upon request).

#### Embryo mortality and fitness parameters

Embryo mortality and fitness measures were taken from the three captive populations held at the Max Planck Institute for Ornithology (“Seewiesen”, “Bielefeld”, and “Cracow”). Eggs were classified as infertile, naturally died embryo, hatched but died <35 days of age, and independent offspring (≥35 days of age) as described in Ihle et al. [107]. Data were collected in four different experimental set-ups (Table 3): (1) cage laying, representing pairs that were allowed to lay eggs in isolated cages, and whose eggs were removed after four to five days, or were cross-fostered, or whose offspring were sacrificed right after hatching. For these pairs we analyzed fecundity (i.e., the number of eggs laid including infertile eggs) as a female fitness trait and—if information was available—the embryo mortality rate. (2) Cage breeding, representing pairs that were allowed to lay eggs and raise offspring in isolated cages. We analyzed fecundity (including infertile eggs) as a female fitness trait, the number of fledglings (≥35 days of age) as female and male reproductive success, and the embryo mortality rate. (3) Aviary laying, representing pairs that laid eggs in communal aviaries, and whose eggs were removed after four to five days, or cross-fostered with parentage assigned genetically. We analyzed fecundity (i.e., the number of eggs laid excluding infertile eggs because they cannot be assigned with certainty to the parents) as a female fitness trait, siring success as a male fitness trait and—if information was available—the embryo mortality rate. (4) Aviary breeding, representing pairs that bred in communal aviaries and who raised offspring. We analyzed fecundity (excluding infertile eggs because they cannot be assigned with certainty to the parents) as a female fitness trait, siring success as a male fitness trait, the number of fledglings (≥35 days of age) as female and male reproductive success, and the embryo mortality rate. Sample sizes are given in Table 3.

### Association analyses and software

#### Software

All analyses were performed in R (v3.0.2) [108]. Mixed-effects models were fitted using ASReml-R (v3) [109]. Inbreeding coefficients for each individual were calculated using the pedigreemm package (v0.3-1) [110]. Meta-analyses using a fixed-effect model were done with the meta.summaries() function in the rmeta package (v2.16) [111].

#### Embryo mortality

We fitted mixed-effects generalized linear models with embryo mortality as the binomial response variable (0 = embryo survived until hatch, 1 = embryo died naturally, infertile eggs excluded) and the mother’s and the father’s inversion genotypes as two predictors (underdominance effect coded as 0 = homozygous for either inversion genotype, 1 = heterozygous). We included the pedigree-based inbreeding coefficient of the embryo (as a covariate) and the pair identity and the mother’s and father’s additive genetic relatedness matrices calculated from the pedigree as random effects. We also controlled for experimental setup (cage versus aviary breeding and cage versus aviary laying) by fitting it as a factor with four levels and the specific experiment (43 levels across 12 years and 3 populations) as an additional random effect.

#### Morphological phenotypes

We used the Z-transformed morphological phenotypes as the response variable in univariate mixed-effects linear models and the individual’s inversion genotype simultaneously as an additive effect (coded as −1 = homozygous for the minor allele, 0 = heterozygous, 1 = homozygous for the major allele using one degree of freedom) and a dominance effect (coded as 0 = homozygous for either inversion genotype, 1 = heterozygous) as two predictors.

In the wild “Fowlers Gap” population we included sex (factor with two levels), the individual’s estimated age (covariate for subadults), and season (factor with two levels referring to the catching seasons October–December 2010 and April–May 2011) as fixed effects and for body mass we also included time of day (covariate). We corrected for overall body size by fitting body mass as a covariate (when body mass was the independent variable we used tarsus length instead). We controlled for relatedness by fitting a genetic similarity matrix (GSM) as a random effect (see [112] for details). We first LD-pruned all SNPs (composite LD cutoff of 0.2 across the whole genome) and then used all remaining autosomal SNPs (n = 3138) to construct the GSM as described for “Method 1” in [112].

In the “Sydney” population we used the same fixed effects as in the “Fowlers Gap” analyses and additionally included the observer of the measurement (factor with two levels). Since we had neither pedigree nor genome-wide SNP data for these birds, we did not control for relatedness.

In the three captive populations (“Seewiesen”, “Bielefeld”, and “Cracow”) we fitted the individual’s sex (factor), the observer of the measurement (factor with maximally five levels), the individual’s pedigree-based inbreeding coefficient (covariate), and its known exact age (covariate) as fixed effects. For body mass we included time of day (covariate) and in the “Seewiesen” population we included whether the bird was measured dead or live (factor) for the three beak morphology traits as fixed effects (see [113] for details). We controlled for body size as in the “Fowlers Gap” population. We controlled for relatedness by fitting an additive genetic relatedness matrix calculated from the pedigree as a random effect.

#### Fitness parameters

We fitted univariate mixed-effects linear models using each of the four fitness parameters (female fecundity, male siring success, female reproductive success, male reproductive success) as the dependent variable in the three captive populations and the inversion genotype of the individual coded as an additive (−1 = homozygous for the minor allele, 0 = heterozygous, 1 = homozygous for the major allele using one degree of freedom) and dominance (0 = homozygous for either inversion genotype, 1 = heterozygous) effect as two predictors. We first square root-transformed the dependent variables to improve model fit and Z-transformed them prior to analysis. We also fitted Poisson models, which qualitatively gave the same results (not shown).

As fixed effects we included the pedigree-based inbreeding coefficient of the individual (covariate) and for female fecundity, male siring success, and female and male reproductive success we added the number of days an individual spent in the respective experiment (covariate). We fitted an additive genetic relatedness matrix calculated from the pedigree as a random effect and since we had multiple measures per individual we also fitted a permanent environment random effect. We controlled for experimental setup (cage versus aviary breeding and cage versus aviary laying) by fitting it as a fixed effect (factor with four levels) and the specific experiment as an additional random effect.

#### Permutations and power analyses

To obtain the expected distribution of effect sizes of inversion genotypes on morphological and fitness traits under randomness (null distribution), we permuted inversion genotypes 100 times within each sex, fitted the same mixed models for each inversion including the same fixed and random effects as for the empirical data, and extracted additive and dominance effect size estimates. We then calculated the same meta-analytic summary statistics as for the empirical data.

We estimated the power to detect effects of different magnitude given our data using the following approach. First, we permuted inversion genotypes ten times within each sex and effect size category (10 permutations × 10 effect sizes = 100 simulations). For an additive effect, we then added or subtracted a predefined effect size from the phenotypic values of the two homozygous groups of individuals. For a dominance effect, we added a predefined effect size to the phenotypic values of the heterozygous individuals. We then fitted the same mixed models for each inversion including the same fixed and random effects as for the empirical data, extracted additive and dominance effect size estimates, and calculated the same meta-analytic summary statistics as for the empirical data.

#### Frequency-dependent selection

To test for frequency-dependent selection we used the fitness data (female fecundity, male siring success, female reproductive success, male reproductive success) measured in the aviary setting in two captive populations (“Seewiesen” and “Bielefeld”). For the “Seewiesen” population we had data from 72 aviaries (12 for reproductive success), each with 12–15 birds. For the “Bielefeld” population we had data from 23 aviaries with 10–12 individuals each. Allele frequency ranges are provided in Additional file 2: Table S6. For each aviary we first calculated the inversion allele frequencies (the proportion of inversion alleles A, B, and C in an aviary) considering only birds of the sex in which the fitness parameter under consideration was assessed. Then, for all those individuals in an aviary we calculated the sum of the aviary-specific allele frequencies of its two inversion alleles. For example, given an inversion with only two haplotypes (A and B) and an allele frequency of inversion type A of 0.8 in an aviary, all individuals in that aviary with genotype AA would get a value of 0.8 + 0.8 = 1.6, all individuals with genotype BB 0.2 + 0.2 = 0.4, and heterozygous individuals 0.2 + 0.8 = 1. Thus, in case of an inversion with only two haplotypes (as those on chromosomes *Tgu5*, *Tgu11*, and *Tgu13*), the sum for heterokaryotypic individuals always equals 1 and the two homozygotes deviate equally from 1, unless the two alleles have an equal frequency (additive effect).

We then used this sum as the predictor in univariate mixed-effects linear models using each of the four fitness parameters (female fecundity, male siring success, female reproductive success, and male reproductive success) as the dependent variable in the two captive populations. We first square root-transformed the dependent variables to improve model fit and Z-transformed them prior to analysis. As fixed and random effects we included the same variables as in the other fitness models (see above).

#### Segregation distortion

We tested whether the four inversion polymorphisms were transmitted to the next generation in a fair Mendelian way. For each heterokaryotypic parent we counted how many times it transmitted the major and minor allele to its offspring. We summed the number of transmissions across all families and populations and used the binomial test to assess fair Mendelian segregation of alleles. For the autosomal inversions we either included only heterokaryotypic females, only heterokaryotypic males, or both sexes combined. On chromosome *TguZ* we tested all two-way combinations of the three inversion types in heterokaryotypic males only. See [87] for methodological details.

## Additional files

Additional file 1: Figure S1. Linkage disequilibrium and principal component analysis results along chromosomes *Tgu2*, *Tgu26* and *Tgu27*. **Figure S2.** Composite LD between eigenvectors of PC1 to PC3 and the SNPs along chromosome *Tgu5*. **Figure S3.** Composite LD between eigenvectors of PC1 to PC3 and the SNPs along chromosome *Tgu11*. **Figure S4.** Composite LD between eigenvectors of PC1 to PC4 and the SNPs along chromosome *Tgu13*. **Figure S5.** Composite LD between eigenvectors of PC1 to PC5 and the SNPs along chromosome *TguZ*. **Figure S6.** Median-joining networks of phased SNPs for the inversions on chromosomes *Tgu5*, *Tgu11*, *Tgu13* and *TguZ*. **Figure S7.** Diversity in 50 kb windows along each chromosome in the zebra finch genome. **Figure S8.** Dominance effects of mother’s and father’s inversion karyotype on embryo mortality in three captive populations. **Figure S9.** Additive effects of the minor inversion allele on different fitness parameters in three captive populations. **Figure S10.** Negative frequencydependent selection effects on different fitness parameters in two captive populations. **Figure S11.** Dominance effects of the minor inversion allele on morphological phenotypes in three captive and two wild populations. **Figure S12.** Composite LD along chromosome *TguZ* between all combinations of the three inversion haplotypes. **Figure S13.** Linkage disequilibrium and principal component analysis results along chromosomes *Tgu5*, *Tgu11*, *Tgu13* and *TguZ* using a filtered SNP set. **Figure S14.** Principle component analysis results from the wild “Fowlers Gap” birds along chromosome *Tgu5*, *Tgu11*, *Tgu13* and *TguZ* together with the founders of the three captive populations. Color coded are the inversion type calls for each individual using only the information from the tag SNPs as described in the Methodssection. **Figure S15.** Principle component analysis results from the wild “Fowlers Gap” birds along chromosome *Tgu5*, *Tgu11*, *Tgu13* and *TguZ* with PCA scores of founder individuals of the three captive populations overlaid. (DOCX 8865 kb) Additional file 2: Table S1. Estimates of composite LD between inversions in wild Australian zebra finches. **Table S2.** Transmission ratios of each inversion polymorphism combining data of all three captive populations ("Seewiesen", "Bielefeld", "Cracow"). **Table S3.** Description of the 15 tag SNPs. **Table S4.** Comparison between Sequenom calling of inversion genotypes using the tag SNPs and Illumina calling of inversion types using PCA of all SNPs genotyped on the respective chromosome in "Seewiesen", "Bielefeld", and "Cracow" founder individuals. **Table S5.** Inversion allele frequencies in the four populations of the follow-up study. **Table S6.** Number of aviaries and maximal allele frequency ranges per aviary and inversion that were available for testing frequency-dependent selection. (DOCX 42 kb)

## Abbreviations

H_p_: Pooled heterozygosity
HWE: Hardy–Weinberg equilibrium
IKMB: Institute of Clinical Molecular Biology
IQR: Interquartile range
kb: Kilobases
LD: Linkage disequilibrium
MAC: Minor allele count frequency
Mb: Megabases
OR: Odds ratio
PC: Principle component
PCA: Principal component analysis
SE: Standard error
SNP: Single nucleotide polymorphism
ZH_p_: Z-transformed pooled heterozygosity
