# Supplementary material for: Fitness consequences of polymorphic inversions in the zebra finch genome
Source: Genome Biol. 2016 Sep 29;17:199. doi: 10.1186/s13059-016-1056-3 (PMC5043542; doi:10.1186/s13059-016-1056-3)
Supplement: Additional file 1: Figure S1. — Linkage disequilibrium and principal component analysis results along chromosomes Tgu2, Tgu26 and Tgu27. Figure S2. Composite LD between eigenvectors of PC1 to PC3 and the SNPs along chromosome Tgu5. Figure S3. Composite LD between eigenvectors of PC1 to PC3 and the SNPs along chromosome Tgu11. Figure S4. Composite LD between eigenvectors of PC1 to PC4 and the SNPs along chromosome Tgu13. Figure S5. Composite LD between eigenvectors of PC1 to PC5 and the SNPs along chromosome TguZ. Figure S6. Median-joining networks of phased SNPs for the inversions on chromosomes Tgu5, Tgu11, Tgu13 and TguZ. Figure S7. Diversity in 50 kb windows along each chromosome in the zebra finch genome. Figure S8. Dominance effects of mother’s and father’s inversion karyotype on embryo mortality in three captive populations. Figure S9. Additive effects of the minor inversion allele on different fitness parameters in three captive populations. Figure S10. Negative frequencydependent selection effects on different fitness parameters in two captive populations. Figure S11. Dominance effects of the minor inversion allele on morphological phenotypes in three captive and two wild populations. Figure S12. Composite LD along chromosome TguZ between all combinations of the three inversion haplotypes. Figure S13. Linkage disequilibrium and principal component analysis results along chromosomes Tgu5, Tgu11, Tgu13 and TguZ using a filtered SNP set. Figure S14. Principle component analysis results from the wild “Fowlers Gap” birds along chromosome Tgu5, Tgu11, Tgu13 and TguZ together with the founders of the three captive populations. Color coded are the inversion type calls for each individual using only the information from the tag SNPs as described in the Methodssection. Figure S15. Principle component analysis results from the wild “Fowlers Gap” birds along chromosome Tgu5, Tgu11, Tgu13 and TguZ with PCA scores of founder individuals of the three captive populations overlaid. (DOCX 8865 kb) [file 13059_2016_1056_MOESM1_ESM.docx]

**Additional file 1**

**Figure S1:** The left panels depict linkage disequilibrium (LD) and the right panels principal component analysis results along chromosomes *Tgu2* (**A**, **B**), *Tgu26* (**C**, **D**) and *Tgu27* (**E**, **F**). Above each LD plot marker positions in Mb are given. PCA included all SNPs on the respective chromosome. Chromosome *Tgu2* (n = 539 SNPs) is shown as an example where linkage blocks are lacking (except for a small region around the centromere around 82 Mb).

**Figure S2:** Composite LD between the eigenvectors of PC1 to PC3 and the SNPs along chromosome *Tgu5* as the squared Pearson’s correlation coefficient. SNPs within the inversion are highlighted in color. For each principle component the percentage of variance explained is indicated.

**Figure S3:** Composite LD between the eigenvectors of PC1 to PC3 and the SNPs along chromosome *Tgu11* as the squared Pearson’s correlation coefficient. SNPs within the inversion are highlighted in color. For each principle component the percentage of variance explained is indicated.

**Figure S4:** Composite LD between the eigenvectors of PC1 to PC4 and the SNPs along chromosome *Tgu13* as the squared Pearson’s correlation coefficient. SNPs within the inversion are highlighted in color. For each principle component the percentage of variance explained is indicated.

**Figure S5:** Composite LD between the eigenvectors of PC1 to PC5 and the SNPs along chromosome *TguZ* as the squared Pearson’s correlation coefficient. SNPs within the inversion are highlighted in color. For each principle component the percentage of variance explained is indicated.

**Figure S6:** Median-joining networks of (**A**) the phased SNPs in the presumed breakpoint regions of chromosome *Tgu5*, (**B**) all phased SNPs within the inverted region on chromosome *Tgu11* (there were too few SNPs at the breakpoint), (**C**) the phased SNPs in the presumed breakpoint regions of chromosome *Tgu13*, and (**D**) all SNPs within the inverted region on chromosome *TguZ* in females (breakpoint regions are unclear on chromosome *TguZ*). For each chromosome 10% (n = 188) of all haplotypes are shown and the haplotypes are named as in Table 2.

**Figure S7:** (**A**) Diversity (SNPs per site) in 50 kb windows along each chromosome in the zebra finch genome. Highlighted areas are the presumed inversion breakpoints on the autosomes and the entire inversion interior on the sex chromosome. The SNPs per site at the breakpoints (B), the inversion interior (I) and outside the inversion (O) are depicted for chromosome *Tgu5* (**B**), *Tgu11* (**C**), *Tgu13* (**D**) and *TguZ* (**E**).

**Figure S8:** Dominance effects (Odds ratio ± 95% confidence intervals) of mother’s and father’s inversion karyotype on embryo mortality in three captive zebra finch populations (S = ”Seewiesen”, B = ”Bielefeld”, C = ”Cracow” and M = meta-analytic summary). An odds ratio > 1 indicates an increased rate of embryo mortality in the offspring of females (top row) or males (bottom row) that are heterozygous for one of the four inversions on chromosomes *Tgu5*, *Tgu11*, *Tgu13* and *TguZ*. The point sizes reflect log-transformed sample sizes.

**Figure S9:** Additive effects of the minor inversion allele (± 95% confidence intervals) on different fitness parameters (RS = reproductive success) in three captive zebra finch populations (S = ”Seewiesen”, B = ”Bielefeld”, C = ”Cracow” and M = meta-analytic summary). The point size reflects log-transformed sample sizes. None of the meta-analytic summary estimates survived strict Bonferroni correction.

**Figure S10:** Negative frequency-dependent selection effects ± 95% confidence intervals on different fitness parameters (RS = reproductive success) in two captive zebra finch populations (S = ”Seewiesen”, B = ”Bielefeld” and M = meta-analytic summary). A negative effect means that rare alleles have higher fitness. The point size reflects log-transformed sample sizes. None of the meta-analytic summary estimates survived strict Bonferroni correction.

**Figure S11:** Heterotic (dominance) effects of the minor inversion allele (± 95% confidence intervals) on morphological phenotypes in three captive and two wild zebra finch populations (S = ”Seewiesen”, B = ”Bielefeld”, C = ”Cracow”, Sy = “Sydney”, W = “Fowlers Gap” and M = meta-analytic summary). The point size reflects log-transformed sample sizes. None of the meta-analytic summary estimates survived strict Bonferroni correction.

**Figure S12:** Composite linkage disequilibrium (LD) along chromosome *TguZ*. LD was calculated for the subset of birds that were (A) homozygote / hemizygote for inversion types A or B or heterozygote AB (n = 771 individuals), (B) homozygote / hemizygote for inversion types A or C or heterozygote AC (n = 477 individuals) and (C) homozygote / hemizygote for inversion types B or C or heterozygote BC (n = 243 individuals). Inversion types are named as in Figure 1. Above the LD plots marker positions in Mb are given.

**Figure S13:** The left panel depicts linkage disequilibrium (LD) and the right panel principal component analysis (PCA) results along chromosomes *Tgu5* (**A**, **B**), *Tgu11* (**C**, **D**), *Tgu13* (**E**, **F**) and *TguZ* (**G**, **H**). Above the LD plots marker positions in Mb are given. SNPs had been filtered prior to analyses using the “earliest finish time” greedy algorithm to include only those SNPs that were separated by minimally 185 kb.

**Figure S14:** Principle component analysis (PCA) results from the wild “Fowlers Gap” birds along chromosome *Tgu5* (**A**), *Tgu11* (**B**), *Tgu13* (**C**), *TguZ* (**D**). Founders of the “Seewiesen”, “Bielefeld” and “Cracow” population (n = 239 individuals) were run also on the Illumina genotyping platform and we used the SNP-loadings on PC1 and PC2 from the PCA of the “Fowlers Gap” birds on these birds to calculate a PCA score for each individual. Then we called the inversion types for each individual (“Fowlers Gap”, “Seewiesen”, “Bielefeld” and “Cracow” population) using only the information from the tag SNPs as described in the Methods section. The inversion type calling using the tag SNPs is color coded. Using a lenient majority vote decision rule, all individuals were correctly assigned to their haplotype clusters on chromosomes *Tgu5*, *Tgu11* and *Tgu13*. Using a more strict unanimity decision rule on chromosome *TguZ* for the “Fowlers Gap” and “Bielefeld” birds, inversion genotypes from the tag SNPs correspond perfectly with the PCA results, but leave some individuals uncalled.

**Figure S15:** Principle component analysis (PCA) results from the wild “Fowlers Gap” birds along chromosome *Tgu5* (**A**), *Tgu11* (**B**), *Tgu13* (**C**), *TguZ* (**D**). Founders of the “Seewiesen”, “Bielefeld” and “Cracow” population (n = 239 individuals) were also run on the Illumina genotyping platform and we used the SNP-loadings on PC1 and PC2 from the PCA of the “Fowlers Gap” birds on these birds to calculate a PCA score for each individual. They are added in different colors to the figure. All founders reside within the clusters formed by the “Fowlers Gap” birds. However, note that in the Bielefeld population on chromosome *TguZ* a “recombinant” haplotype exists (between the CC homozygous and BC heterokaryotypic individuals).
