# Supplementary material for: Fitness consequences of polymorphic inversions in the zebra finch genome
Source: Genome Biol. 2016 Sep 29;17:199. doi: 10.1186/s13059-016-1056-3 (PMC5043542; doi:10.1186/s13059-016-1056-3)
Supplement: Additional file 2: Table S1. — Estimates of composite LD between inversions in wild Australian zebra finches. Table S2. Transmission ratios of each inversion polymorphism combining data of all three captive populations ("Seewiesen", "Bielefeld", "Cracow"). Table S3. Description of the 15 tag SNPs. Table S4. Comparison between Sequenom calling of inversion genotypes using the tag SNPs and Illumina calling of inversion types using PCA of all SNPs genotyped on the respective chromosome in "Seewiesen", "Bielefeld", and "Cracow" founder individuals. Table S5. Inversion allele frequencies in the four populations of the follow-up study. Table S6. Number of aviaries and maximal allele frequency ranges per aviary and inversion that were available for testing frequency-dependent selection. (DOCX 42 kb) [file 13059_2016_1056_MOESM2_ESM.docx]

**Additional file 2**

**Table S1:** Estimates of composite LD between inversions in wild Australian zebra finches. LD between autosomes and chromosome *TguZ* was estimated by including the chromosome *TguW* in females and then using the method described in [1] to obtain r^2^ and P-values for loci with multiple alleles.

| **Chromosomes** | **Genotype** | **Genotype counts** | | **Composite LD (r^2^)** | **P** |
| --- | --- | --- | --- | --- | --- |
|  |  | **males** | **females** |  |  |
| *Tgu5 – Tgu11* | AA – AA | 49 | 62 | 0.0031 | 0.087 |
|  | AA – AB | 77 | 88 |  |  |
|  | AA – BB | 28 | 42 |  |  |
|  | AB – AA | 56 | 55 |  |  |
|  | AB – AB | 123 | 94 |  |  |
|  | AB – BB | 53 | 55 |  |  |
|  | BB – AA | 19 | 26 |  |  |
|  | BB – AB | 45 | 36 |  |  |
|  | BB – BB | 18 | 22 |  |  |
| *Tgu5 – Tgu13* | AA – AA | 33 | 52 | 0.00028 | 0.60 |
|  | AA – AB | 88 | 94 |  |  |
|  | AA – BB | 33 | 46 |  |  |
|  | AB – AA | 74 | 56 |  |  |
|  | AB – AB | 112 | 100 |  |  |
|  | AB – BB | 46 | 48 |  |  |
|  | BB – AA | 22 | 20 |  |  |
|  | BB – AB | 43 | 44 |  |  |
|  | BB – BB | 17 | 20 |  |  |
| *Tgu5 – TguZ* | AA – AA / AW | 44 | 105 | 0.0013 | 0.33 |
|  | AA – AB | 58 | 0 |  |  |
|  | AA – BB / BW | 14 | 62 |  |  |
|  | AA – BC | 9 | 0 |  |  |
|  | AA – CC / CW | 3 | 12 |  |  |
|  | AA – AC | 6 | 0 |  |  |
|  | AB – AA / AW | 70 | 117 |  |  |
|  | AB – AB | 82 | 0 |  |  |
|  | AB – BB / BW | 16 | 65 |  |  |
|  | AB – BC | 8 | 0 |  |  |
|  | AB – CC / CW | 0 | 11 |  |  |
|  | AB – AC | 28 | 0 |  |  |
|  | BB – AA / AW | 26 | 44 |  |  |
|  | BB – AB | 34 | 0 |  |  |
|  | BB – BB / BW | 6 | 28 |  |  |
|  | BB – BC | 2 | 0 |  |  |
|  | BB – CC / CW | 1 | 6 |  |  |
|  | BB – AC | 4 | 0 |  |  |
| *Tgu11 – Tgu13* | AA – AA | 40 | 37 | 0.00050 | 0.49 |
|  | AA – AB | 57 | 74 |  |  |
|  | AA – BB | 27 | 32 |  |  |
|  | AB – AA | 63 | 64 |  |  |
|  | AB – AB | 130 | 103 |  |  |
|  | AB – BB | 52 | 51 |  |  |
|  | BB – AA | 26 | 27 |  |  |
|  | BB – AB | 56 | 61 |  |  |
|  | BB – BB | 17 | 31 |  |  |
| *Tgu11 – TguZ* | AA – AA / AW | 42 | 85 | 0.0024 | 0.10 |
|  | AA – AB | 42 | 0 |  |  |
|  | AA – BB / BW | 8 | 43 |  |  |
|  | AA – BC | 5 | 0 |  |  |
|  | AA – CC / CW | 0 | 6 |  |  |
|  | AA – AC | 10 | 0 |  |  |
|  | AB – AA / AW | 75 | 119 |  |  |
|  | AB – AB | 88 | 0 |  |  |
|  | AB – BB / BW | 17 | 70 |  |  |
|  | AB – BC | 12 | 0 |  |  |
|  | AB – CC / CW | 4 | 15 |  |  |
|  | AB – AC | 24 | 0 |  |  |
|  | BB – AA / AW | 23 | 62 |  |  |
|  | BB – AB | 44 | 0 |  |  |
|  | BB – BB / BW | 11 | 42 |  |  |
|  | BB – BC | 2 | 0 |  |  |
|  | BB – CC / CW | 0 | 8 |  |  |
|  | BB – AC | 4 | 0 |  |  |
| *Tgu13 – TguZ* | AA – AA / AW | 33 | 71 | 0.0019 | 0.18 |
|  | AA – AB | 53 | 0 |  |  |
|  | AA – BB / BW | 7 | 44 |  |  |
|  | AA – BC | 7 | 0 |  |  |
|  | AA – CC / CW | 2 | 8 |  |  |
|  | AA – AC | 14 | 0 |  |  |
|  | AB – AA / AW | 74 | 126 |  |  |
|  | AB – AB | 89 | 0 |  |  |
|  | AB – BB / BW | 23 | 81 |  |  |
|  | AB – BC | 8 | 0 |  |  |
|  | AB – CC / CW | 2 | 16 |  |  |
|  | AB – AC | 17 | 0 |  |  |
|  | BB – AA / AW | 33 | 69 |  |  |
|  | BB – AB | 32 | 0 |  |  |
|  | BB – BB / BW | 6 | 30 |  |  |
|  | BB – BC | 4 | 0 |  |  |
|  | BB – CC / CW | 0 | 5 |  |  |
|  | BB – AC | 7 | 0 |  |  |

**Table S2:** Transmission ratios of each inversion polymorphism combining data of all three captive populations (“Seewiesen”, “Bielefeld”, “Cracow”) in heterokaryotypic mothers (including cases where their male partner was also heterokaryotypic), heterokaryotypic fathers (including cases where their female partner was also heterokaryotypic) and both sexes combined. For each inversion the number of transmissions of the major allele was tested against the number of transmissions of the minor allele (types A *vs* B). On chromosome *TguZ* we tested types A *vs* B, A *vs* C and B *vs* C. E(n_A_) and n_A_ are the expected and observed numbers of inheritance events of the major allele.

| **Chromosome** | **Scan** | **Informative meioses** | **n_A_** | **E(n_A_)** | **Transmission ratio** | **95% CI** | **P** |
| --- | --- | --- | --- | --- | --- | --- | --- |
| *Tgu5* | Female | 2435 | 1221 | 1217.5 | 0.501 | 0.481, 0.521 | 0.90 |
| *Tgu5* | Male | 2626 | 1298 | 1313 | 0.494 | 0.475, 0.514 | 0.57 |
| *Tgu5* | Combined | 3533 | 1765 | 1766.5 | 0.500 | 0.483, 0.516 | 0.97 |
| *Tgu11* | Female | 2826 | 1404 | 1413 | 0.497 | 0.478, 0.515 | 0.75 |
| *Tgu11* | Male | 2899 | 1445 | 1449.5 | 0.498 | 0.480, 0.517 | 0.88 |
| *Tgu11* | Combined | 3865 | 1908 | 1932.5 | 0.494 | 0.478, 0.510 | 0.44 |
| *Tgu13* | Female | 2216 | 1079 | 1108 | 0.487 | 0.466, 0.508 | 0.23 |
| *Tgu13* | Male | 1986 | 988 | 993 | 0.497 | 0.475, 0.520 | 0.84 |
| *Tgu13* | Combined | 3038 | 1486 | 1519 | 0.489 | 0.471, 0.507 | 0.24 |
| *TguZ* – A vs B | Male | 1142 | 557 | 571 | 0.488 | 0.458, 0.517 | 0.42 |
| *TguZ* – A vs C | Male | 832 | 420 | 416 | 0.505 | 0.470, 0.539 | 0.81 |
| *TguZ* – B vs C | Male | 483 | 234 | 241.5 | 0.484 | 0.439, 0.530 | 0.52 |

**Table S3:** Description of the 15 tag SNPs. r^2^ is the composite LD with the inversion genotype. Type A, Type B and Type C indicate the number of A alleles in a homozygous individual for inversion genotypes A, B and C, respectively.

| **Chromosome** | **SNP ID** | **Position** | **r^2^** | **A allele** | **B allele** | **Type A** | **Type B** | **Type C** |
| --- | --- | --- | --- | --- | --- | --- | --- | --- |
| *Tgu5* | WZF00167975 | 1,000,220 | 1 | A | C | 2 | 0 |  |
|  | WZF00170082 | 1,778,553 | 1 | A | C | 0 | 2 |  |
|  | WZF00169329 | 14,526,432 | 1 | T | G | 2 | 0 |  |
| *Tgu11* | WZF00031778 | 12,252,712 | 0.972 | A | G | 2 | 0 |  |
|  | WZF00031788 | 12,268,156 | 0.901 | A | G | 2 | 0 |  |
|  | WZF00031805 | 12,289,339 | 0.994 | T | C | 2 | 0 |  |
| *Tgu13* | WZF00041460 | 171,215 | 1 | A | G | 0 | 2 |  |
|  | WZF00040683 | 11,363,650 | 0.996 | T | C | 2 | 0 |  |
|  | WZF00040731 | 11,536,148 | 0.998 | T | C | 2 | 0 |  |
| *TguZ* | WZF00231991 | 5,954,002 | 1 | A | G | 2 | 0 | 2 |
|  | WZF00218433 | 37,400,431 | 0.986 | T | C | 2 | 2 | 0 |
|  | WZF00218859 | 38,265,467 | 1 | A | C | 0 | 2 | 0 |
|  | WZF00222123 | 44,773,107 | 0.986 | T | C | 2 | 2 | 0 |
|  | WZF00223156 | 46,527,787 | 0.986 | A | G | 0 | 2 | 2 |
|  | WZF00237790 | 65,844,335 | 0.986 | A | G | 0 | 2 | 2 |

**Table S4:** Comparison between Sequenom calling of inversion genotypes using the tag SNPs and Illumina calling of inversion types using PCA of all SNPs genotyped on the chromosome in n = 127 Seewiesen, n = 74 Bielefeld and n = 25 Cracow founder individuals. There were additional 10 Fowlers Gap individuals. 1,062 Seewiesen individuals had been previously genotyped with 37 SNPs on chromosome *TguZ* and we compared the calling between Sequenom and the PCA results using these 37 SNPs.

| **Chromosome** | **n Errors** | **n Uncalled** | **n Comparisons** | **Assay** | **Calling algorithm** |
| --- | --- | --- | --- | --- | --- |
| *Tgu5* | 0 | 0 | 234 | Illumina iSelect (same SNPs) | Majority vote |
| *Tgu11* | 0 | 0 | 236 | Illumina iSelect (same SNPs) | Majority vote |
| *Tgu13* | 0 | 0 | 236 | Illumina iSelect (same SNPs) | Majority vote |
| *TguZ* | 0 | 2 | 200 | Illumina iSelect (same SNPs) | Majority vote; in Bielefeld restrict calling to individuals where all SNPs are genotyped and fit, which removes 26 individuals (“recombinants”) |
| *TguZ* | 0 | 0 | 1062 | Illumina GoldenGate (different SNPs) | Majority vote |

**Table S5:** Inversion allele frequencies in the four populations of the follow-up study.

| **Population** | ***Tgu5*** | |  | ***Tgu11*** | |  | ***Tgu13*** | |  | ***TguZ*** | | |
| --- | --- | --- | --- | --- | --- | --- | --- | --- | --- | --- | --- | --- |
|  | **A** | **B** |  | **A** | **B** |  | **A** | **B** |  | **A** | **B** | **C** |
| **Seewiesen** | 0.714 | 0.286 |  | 0.492 | 0.508 |  | 0.724 | 0.276 |  | 0.409 | 0.338 | 0.253 |
| **Bielefeld** | 0.248 | 0.752 |  | 0.324 | 0.676 |  | 0.79 | 0.21 |  | 0.822 | 0.178 | 0 |
| **Cracow** | 0.675 | 0.325 |  | 0.54 | 0.46 |  | 0.52 | 0.48 |  | 0.546 | 0.272 | 0.181 |
| **Sydney** | 0.608 | 0.392 |  | 0.445 | 0.555 |  | 0.515 | 0.485 |  | 0.613 | 0.304 | 0.083 |

**Table S6:** Number of aviaries and maximal allele frequency ranges per aviary and inversion that were available for testing frequency-dependent selection in the “Seewiesen” and “Bielefeld” population. There were 12–15 individuals in an aviary in the “Seewiesen” population and 10–12 individuals in an aviary in the “Bielefeld” population.

| **Population** | **Parameter** | **Chromosome** | **# Aviaries** | **Frequency range of inversion type** | | |
| --- | --- | --- | --- | --- | --- | --- |
|  |  |  |  | **A** | **B** | **C** |
| Seewiesen | Female fecundity | *Tgu5* | 72 | 0.333–1.000 | 0.000–0.667 |  |
|  | Female fecundity | *Tgu11* | 72 | 0.167–0.833 | 0.167–0.833 |  |
|  | Female fecundity | *Tgu13* | 72 | 0.417–1.000 | 0.000–0.583 |  |
|  | Female fecundity | *TguZ* | 72 | 0.000–0.833 | 0.000–0.833 | 0.000–0.833 |
|  | Male siring success | *Tgu5* | 72 | 0.417–1.000 | 0.000–0.583 |  |
|  | Male siring success | *Tgu11* | 72 | 0.167–0.889 | 0.111–0.833 |  |
|  | Male siring success | *Tgu13* | 72 | 0.417–1.000 | 0.000–0.583 |  |
|  | Male siring success | *TguZ* | 72 | 0.083–0.750 | 0.167–0.583 | 0.000–0.583 |
|  | Female reproductive success | *Tgu5* | 12 | 0.333–0.917 | 0.083–0.667 |  |
|  | Female reproductive success | *Tgu11* | 12 | 0.167–0.750 | 0.250–0.833 |  |
|  | Female reproductive success | *Tgu13* | 12 | 0.583–1.000 | 0.000–0.417 |  |
|  | Female reproductive success | *TguZ* | 12 | 0.000–0.667 | 0.000–0.833 | 0.000–0.833 |
|  | Male reproductive success | *Tgu5* | 12 | 0.500–0.833 | 0.167–0.500 |  |
|  | Male reproductive success | *Tgu11* | 12 | 0.250–0.750 | 0.250–0.750 |  |
|  | Male reproductive success | *Tgu13* | 12 | 0.500–1.000 | 0.000–0.500 |  |
|  | Male reproductive success | *TguZ* | 12 | 0.167–0.667 | 0.167–0.500 | 0.000–0.583 |
| Bielefeld | Female fecundity | *Tgu5* | 23 | 0.000–0.500 | 0.500–1.000 |  |
|  | Female fecundity | *Tgu11* | 23 | 0.083–0.600 | 0.400–0.917 |  |
|  | Female fecundity | *Tgu13* | 23 | 0.500–1.000 | 0.000–0.500 |  |
|  | Female fecundity | *TguZ* | 23 | 0.250–1.000 | 0.000–0.750 | 0.000–0.000 |
|  | Male siring success | *Tgu5* | 23 | 0.083–0.500 | 0.500–0.917 |  |
|  | Male siring success | *Tgu11* | 23 | 0.083–0.500 | 0.500–0.917 |  |
|  | Male siring success | *Tgu13* | 23 | 0.417–0.917 | 0.083–0.583 |  |
|  | Male siring success | *TguZ* | 23 | 0.625–1.000 | 0.000–0.375 | 0.000–0.000 |
|  | Female reproductive success | *Tgu5* | 23 | 0.000–0.500 | 0.500–1.000 |  |
|  | Female reproductive success | *Tgu11* | 23 | 0.083–0.600 | 0.400–0.917 |  |
|  | Female reproductive success | *Tgu13* | 23 | 0.500–1.000 | 0.000–0.500 |  |
|  | Female reproductive success | *TguZ* | 23 | 0.250–1.000 | 0.000–0.750 | 0.000–0.000 |
|  | Male reproductive success | *Tgu5* | 23 | 0.083–0.500 | 0.500–0.917 |  |
|  | Male reproductive success | *Tgu11* | 23 | 0.083–0.500 | 0.500–0.917 |  |
|  | Male reproductive success | *Tgu13* | 23 | 0.417–0.917 | 0.083–0.583 |  |
|  | Male reproductive success | *TguZ* | 23 | 0.625–1.000 | 0.000–0.375 | 0.000–0.000 |

**REFERENCES**

1. Zaykin DV, Pudovkin A, Weir BS: **Correlation-based inference for linkage disequilibrium with multiple alleles.** *Genetics* 2008, **180:**533–545.
